# Safety and Exploratory Pre–Post Changes Associated with an Orally Disintegrating Three-Strain Probiotic Tablet in Older Adults with Functional Constipation: A Single-Center, Open-Label, Single-Arm Study

**DOI:** 10.3390/diseases14090317

**Published:** 2026-08-31

**Authors:** Daisuke Asaoka, Tsutomu Takeda, Yasuhisa Jimbo, Eiji Kamba, Yusuke Nomoto, Osamu Nomura, Daiki Abe, Kumiko Ueda, Hiroya Ueyama, Hiroyuki Isayama, Mariko Hojo, Akihito Nagahara

**Affiliations:** 1 Department of Gastroenterology, Juntendo Tokyo Koto Geriatric Medical Center, Faculty of Medicine, Juntendo University, Tokyo 136-0075, Japan; 2 Department of Gastroenterology, Faculty of Medicine, Juntendo University, Tokyo 113-8421, Japan

**Keywords:** older adults, functional constipation, probiotics, orally disintegrating tablet, stool form, gut microbiota, quality of life, proton pump inhibitor

## Abstract

Background/Objectives: Functional constipation is common in older adults and impairs quality of life. Evidence for multi-strain orally disintegrating (OD) probiotic formulations in this population remains limited. Methods: In this single-center, open-label, single-arm pre–post study, adults aged 65–90 years meeting the Rome IV criteria for functional constipation were administered an OD tablet containing *Bacillus subtilis* TO-A, *Enterococcus faecium* T-110, and *Clostridium butyricum* TO-A for 8 weeks. The primary endpoint was change in the defecation score based on a modified Bristol Stool Form Scale. Secondary endpoints included stool frequency, straining, JPAC-QOL, the modified Constipation Scoring System, Izumo Scale, Dietary Variety Score (DVS), selected metabolic/nutritional variables, and exploratory gut microbiota analyses. Results: Fifty participants provided consent, and 49 were included in the full analysis set; 45 participants completed the 8-week intervention and were included in the paired primary endpoint analysis. The defecation score showed a nominally significant change toward normalization of stool form at week 8 (IQR: −1.0, 7.0; 95%CI: −9.6, −4.2). Stool frequency did not change significantly, whereas constipation-related quality of life and symptom burden significantly changed. DVS increased modestly, and HbA1c decreased slightly; however, these secondary findings should be interpreted as exploratory because no adjustment for multiplicity was performed. Four patients withdrew from this study: one was due to death, but a causal relationship with the study drug was not confirmed. Two gastrointestinal adverse events could have been related to the study product. Exploratory subgroup analyses suggested a larger change in participants not receiving acid-suppressive therapy. Conclusions: The three-strain OD probiotic tablet was associated with changes in stool form and constipation-related symptom burden in older adults with functional constipation. Because this was a single-arm, open-label study without multiplicity adjustment for secondary endpoints, the findings should be regarded as exploratory and hypothesis-generating. Given the absence of a control group, causal efficacy cannot be inferred, and placebo effects, natural symptom fluctuation, or other time-varying confounders cannot be excluded; therefore, the most robust conclusion is that the preparation appeared safe and well tolerated for over 8 weeks in this population.

## 1. Introduction

Chronic constipation is common in older adults and can adversely affect not only bowel habits but also quality of life and daily functioning [1]. In this population, constipation often coexists with frailty, multimorbidity, and polypharmacy, making it an important issue in both geriatric and gastroenterological practice [2]. Safe, practical, and well-tolerated treatment options are therefore needed in routine clinical care.

Current guidelines recommend several pharmacological treatments for chronic constipation, including osmotic laxatives, epithelial secretagogues, and bile acid transporter inhibitors [3]. Probiotics are also widely used in clinical practice and have shown beneficial effects on bowel habits in some studies; however, the magnitude and consistency of their effects vary according to strain composition, formulation, and treatment duration [4,5]. Evidence specific to older adults remains limited, and clinical data addressing potential mechanisms is still scarce.

A three-strain orally disintegrating tablet containing *Clostridium butyricum* TO-A, *Enterococcus faecium* T-110, and *Bacillus subtilis* TO-A is of interest because these bacterial species may have complementary effects on the intestinal environment and gut motility [6,7]. *C. butyricum* has been linked to butyrate-related intestinal homeostasis [8], whereas formulations containing *Bacillus* and *Enterococcus* species may help maintain microbial balance and support broader probiotic effects [9]. Increasingly, evidence has highlighted the gut microbiota’s involvement in systemic diseases and suggested a potential role in regulating host glucose metabolism [10]. However, few prospective studies have evaluated this formulation in older adults while simultaneously assessing stool form, constipation-related quality of life, upper gastrointestinal symptom burden, dietary diversity, glucose metabolism, and gut microbiota profiles.

The present study therefore aimed to evaluate the short-term safety and exploratory 8-week pre–post changes associated with this three-strain orally disintegrating tablet in older adults with functional constipation. In addition to a stool form-based primary endpoint, we assessed constipation symptom burden, quality of life, upper gastrointestinal symptoms, dietary diversity, glucose metabolism, and exploratory changes in the gut microbiota. Because acid-suppressive therapy is increasingly recognized as associated with altered intestinal ecology and oral-to-gut microbial translocation, we also explored whether response patterns differed according to PPI/P-CAB use [11]. This study was designed as a pilot pre–post investigation and was not intended to establish causal efficacy.

## 2. Materials and Methods

### 2.1. Study Design and Participants

This was a single-center, open-label, single-arm pre–post intervention study involving adults aged 65–90 years who met the Rome IV criteria for functional constipation and provided written informed consent [12]. Functional constipation was defined according to the Rome IV criteria as the presence of two or more of the following symptoms:

i. Straining during more than 25% of defecations;
ii. Lumpy or hard stools, corresponding to Bristol Stool Form Scale types 1 or 2, in more than 25% of defecations;
iii. A sensation of incomplete evacuation in more than 25% of defecations;
iv. A sensation of anorectal obstruction or blockage in more than 25% of defecations;
v. Manual maneuvers to facilitate more than 25% of defecations;
vi. Fewer than three spontaneous bowel movements per week.

Participants were required to have symptoms for at least 6 months before enrollment and to fulfill the above criteria during the preceding 3 months.

**Figure 1:**
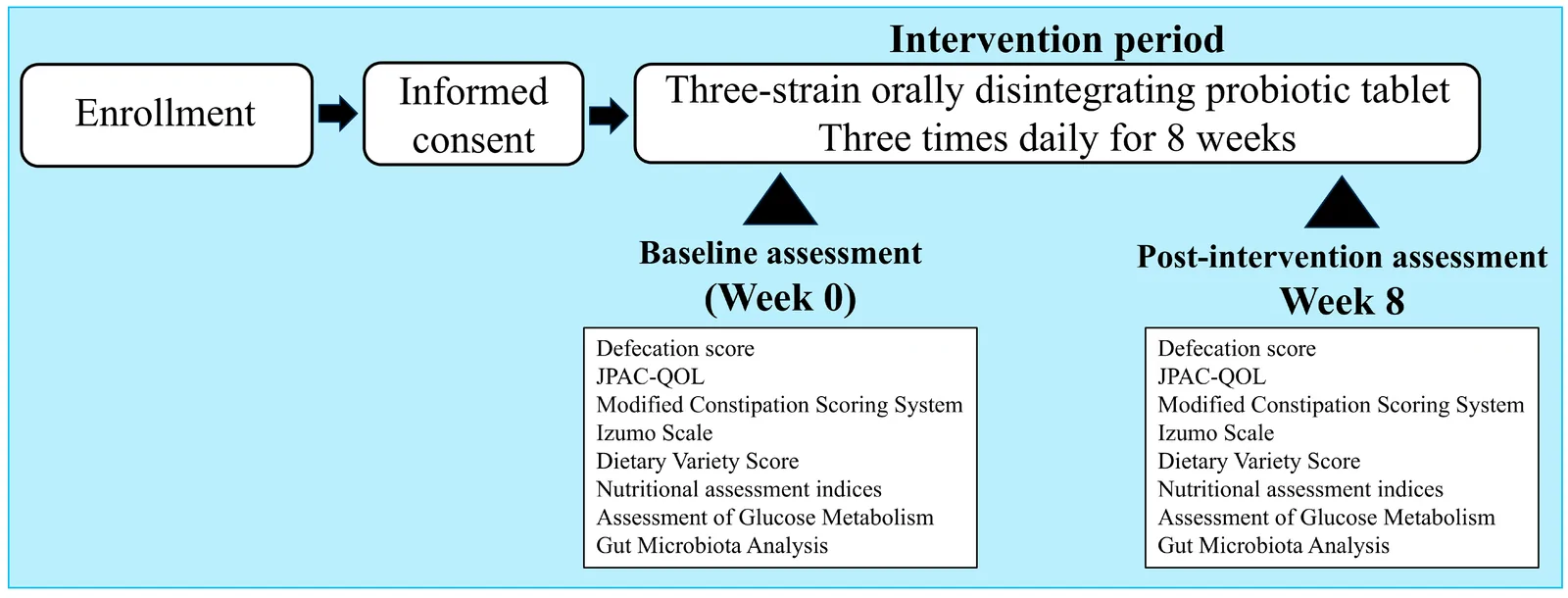
Study protocol.

The study was conducted in accordance with the Declaration of Helsinki, the Clinical Research Act, and relevant enforcement regulations, and was approved by the Juntendo University Certified Review Board (CRB3180012, clinical research plan implementation number (3 August 2021): jRCTs031210344, 22 September 2021). The overall study protocol is shown in Figure 1.

Major exclusion criteria were as follows: 1. met Bristol Stool Form Scale (BSFS) scores of 6 (muddy stool) or 7 (watery stool); 2. a history of surgical resection of the stomach, gallbladder, small intestine, or large intestine (excluding appendicitis and benign polyp removal); 3. a history or current diagnosis of celiac disease, inflammatory bowel disease (Crohn’s disease or ulcerative colitis), or ischemic colitis; 4. intestinal obstruction due to hernia, megacolon, or megarectum; 5. current diagnosis of a malignant digestive system tumor; serious cardiovascular condition; diseases of the respiratory, renal, hepatic, digestive (excluding chronic constipation), hematologic, or nervous system; a psychiatric condition; Parkinson’s disease; or hyper- or hypothyroidism; 6. prior or current participation in clinical, or post-marketing clinical trials, and clinical research for other ethical drugs or medical devices within 3 months before consent was obtained; 7. regular use of medications that affect bowel movements (antibiotics, antidiarrheal drugs) or health foods/supplements (lactic acid bacteria, bifidobacteria, oligosaccharides, dietary fiber, etc.), although cases such as routine yogurt intake were not included; 8. other patients judged inappropriate for study participation by the principal investigator. In addition, participants with organic abnormalities detected by colonoscopy within the previous five years were excluded to reduce the possibility of organic constipation. *H. pylori* infection status was assessed based on medical records obtained as part of routine clinical practice before study registration. Available results of standard diagnostic tests were reviewed, including serum anti-H. pylori antibody testing, the urea breath test, stool antigen testing, rapid urease testing, or histological evaluation using endoscopic biopsy specimens, as well as records of successful eradication therapy. Participants were classified as negative, positive, negative post-eradication, or unknown. *H. pylori* testing was not newly performed at enrollment for all participants.

### 2.2. Study Intervention

Participants received BIO-THREE combination orally disintegrating tablets, each containing *Bacillus subtilis* TO-A 10 mg, *Enterococcus faecium* T-110 2 mg, and *Clostridium butyricum* TO-A 10 mg. Two tablets were taken orally three times daily after meals for 8 weeks.

The potential confounder assessment was limited. Participants were not assigned to a standardized diet, and the quantity or quality of food intake, dietary fiber intake, fluid intake, physical activity level, and seasonal dietary changes were not systematically recorded or controlled. Medication information available from medical records was summarized for selected medication classes, including acid-suppressive therapy, aspirin, prokinetic agents, and laxatives; the use of other probiotic preparations, including intestinal regulators that could confound efficacy assessment and related over-the-counter supplements, was prohibited. However, anticholinergic agents, opioids, antidepressants, antihistamines, minor psychotropic drugs, and other medications that could affect gastrointestinal motility were not comprehensively classified or adjusted for. Colonoscopy was not uniformly repeated immediately before enrollment; therefore, although participants with known organic abnormalities detected by colonoscopy within the previous five years were excluded, undiagnosed polyps, diverticula, active hemorrhoidal disease, or other organic changes could not be completely excluded.

### 2.3. Primary Endpoint

The primary endpoint was the change in defecation score from baseline (week 0) to week 8, calculated from a modified Bristol Stool Form Scale (BSFS) over a 7-day observation period before and after treatment. This endpoint assessed normalization of stool consistency [13].

The BSFS score was recorded daily on a scale from 1 to 7, with a score of 0 assigned on days without defecation. For each day, the daily defecation element score was calculated by subtracting 4, the score corresponding to normal stool consistency, from the daily BSFS score. 

(i) Each daily element is defined as BSFS − 4, so a value of 0 corresponds to the ideal type-4 stool; (ii) a no-defecation day is assigned a BSFS of 0 and a daily element of −4, because a day without any bowel movement is regarded as a state further from normal than even a type-1 stool. This is because it is easy to overlook the passage of hard stools that are difficult to defecate (Types 1 and 2). The total defecation score was defined as the sum of these daily scores over 7 consecutive days. When data were missing for any day during the observation period, the missing value was imputed using the mean of the available daily scores. Values closer to zero indicated normalization toward BSFS type 4; therefore, improvement in this endpoint was interpreted as normalization of stool consistency rather than increased bowel frequency.

In an exploratory analysis, participants were stratified regarding their baseline (week 0) use of acid-suppressive therapy. Participants receiving proton pump inhibitors or potassium-competitive acid blockers were classified as the PPI/P-CAB (+) group, whereas those receiving neither were classified as the PPI/P-CAB (−) group. Changes in subgroup defecation scores from baseline (week 0) to week 8 were assessed using the Wilcoxon signed-rank test. Because these analyses were not prespecified for confirmatory hypothesis testing, the results were interpreted descriptively. 

Participants with missing data within the 7-day period (i.e., those with only 6 days of data) were excluded, and the results are presented as a sensitivity analysis.

### 2.4. Secondary Endpoints

Secondary endpoints included spontaneous bowel movements, straining severity, constipation-specific quality of life, constipation severity, abdominal symptom-related quality of life, dietary variety, nutritional indices, and glucose metabolism markers.

Spontaneous bowel movements were assessed as the 7-day average number of bowel movements. Straining severity was also assessed as the 7-day average and rated on a 5-point scale as follows: 1, no straining; 2, slight straining; 3, moderate straining; 4, strong straining; and 5, very strong straining.

The Bristol Stool Form Scale (BSFS) is a validated visual classification tool that categorizes human stools into seven types according to their form and consistency. Type 1 (separate hard lumps, like nuts) and Type 2 (lumpy, sausage-shaped) indicate constipation; Types 3 (sausage-shaped with surface cracks) and 4 (smooth, soft, sausage- or snake-like) are considered normal; and Types 5 (soft blobs with clear-cut edges), 6 (mushy with ragged edges), and 7 (entirely liquid, no solid pieces) suggest a tendency toward loose stools or diarrhea. The scale serves as a practical surrogate marker of intestinal transit time and is commonly used in both clinical practice and research to assess bowel function. We evaluated changes in bowel movement score on the Bristol Stool Form Scale from baseline (week 0) to week 8 based on patients’ use of acid-suppressive therapy. Participants receiving proton pump inhibitors or potassium-competitive acid blockers were classified as the PPI/P-CAB (+) group, whereas those receiving neither proton pump inhibitors nor potassium-competitive acid blockers were classified as the PPI/P-CAB (−) group.

Constipation-specific quality of life was assessed using the Japanese version of the Patient Assessment of Constipation Quality of Life questionnaire (JPAC-QOL), including the total score and subscale scores [14,15]. The JPAC-QOL consists of 28 items assessing participants’ condition over the previous 2 weeks. Each item is rated on a 5-point scale according to the degree of constipation-related negative impact on quality of life, ranging from 0 (“not at all”) to 4 (“extremely”). The mean score of valid responses is expressed on a scale from 0 to 4, with lower scores indicating better quality of life.

Constipation severity was evaluated using the modified Constipation Scoring System (mCSS), including the total score and each component item [16]. The original CSS consists of eight items: frequency of bowel movements, difficulty of bowel movements, sensation of incomplete evacuation, abdominal pain, time spent in the lavatory per attempt, assistance for evacuation, unsuccessful attempts at evacuation within 24 h, and duration of constipation. In the present study, the mCSS was calculated by excluding the duration of constipation.

Abdominal symptom-related quality of life was assessed using the Izumo Scale, including the total score and each domain score [17]. The Izumo Scale is a self-administered questionnaire designed to quantitatively assess abdominal symptom-related quality of life. It consists of 15 items across five domains: reflux, upper abdominal pain, fullness, constipation, and diarrhea. Each item is scored from 0 to 5 according to symptom severity, with higher scores indicating more severe symptoms. Each domain comprises three items with a score ranging from 0 to 15.

Dietary variety was assessed using the Dietary Variety Score (DVS) [18]. The DVS evaluates the frequency of weekly intake of major food groups commonly consumed in Japan, including meat, seafood, eggs, milk, soybean products, green and yellow vegetables, seaweed, fruits, potatoes, and fats and oils. For each food group, 1 point is assigned if the food is consumed almost every day, and 0 points are assigned if it is consumed less frequently. The total score is calculated as the DVS, with higher scores indicating greater dietary variety.

Nutritional and metabolic indices included the triglyceride to high-density lipoprotein cholesterol ratio (TG/HDL-C ratio) and Geriatric Nutritional Risk Index (GNRI) [19], both of which are utilized to assess the cardiovascular diseases and metabolic syndrome [20] and as the indicator predicting the response to immunotherapy [21], Controlling Nutritional Status (CONUT) score, triglycerides, total cholesterol, and body weight index. The GNRI was calculated using the following formula: GNRI = 14.89 × serum albumin (g/dL) + 41.7 × current body weight (kg)/ideal body weight (kg). The CONUT score was calculated based on serum albumin, total cholesterol, and lymphocyte count as an index reflecting protein metabolism, lipid metabolism, and immune function [22].

Glucose metabolism was assessed using hemoglobin A1c (HbA1c) and C-peptide immunoreactivity (CPR). HbA1c was measured as an index of chronic glycemic status, reflecting average glycemic exposure over the preceding several weeks. CPR was measured as an indicator of endogenous insulin secretion and pancreatic β-cell function. These parameters were evaluated at baseline and after the intervention to assess changes in glucose metabolic status.

### 2.5. Gut Microbiota Analysis

Stool samples were collected at baseline (week 0) and week 8 for gut microbiota analysis using 16S rRNA amplicon sequencing. Alpha diversity was evaluated using the Chao1 richness and Shannon diversity indices. Beta diversity was explored using principal coordinate analysis (PCoA) based on unweighted UniFrac distances.

Gut microbiota composition was analyzed at the Kyoto Institute of Nutrition & Pathology (Kyoto, Japan). Sequencing was performed using the MiSeq platform (Illumina, Tokyo, Japan), and sequence data were processed using Quantitative Insights into Microbial Ecology 2 (QIIME2). Taxonomic assignment was performed using the SILVA database, and results were expressed as relative abundance at the genus level. LEfSe analysis was performed to identify taxa enriched based on time point or acid-suppressive therapy status, using an LDA score > 2.0 on a log10 scale and an unadjusted *p* < 0.05 as exploratory criteria [23]. LEfSe analyses were performed at TOA Biopharma Co., Ltd. (Tokyo, Japan). In these analyses, the class variable was defined by the comparison of interest (time point or PPI/P-CAB status), no subclass variable was used, and within-participant pairing across time points was not modeled. Of the 309 genus-level lineages in the input table, 266 were resolved to the genus level, and the remaining 43 were unresolved at that rank and were retained as unclassified entries at the lowest assigned rank. Because LEfSe also evaluates higher-ranking clades derived from these lineages, a total of 531 clades were subjected to testing in each comparison. No prevalence or abundance filter or correction for multiple comparisons was applied to the relative abundance table. Accordingly, the LEfSe results were regarded as unadjusted rather than confirmatory.

Participants were stratified according to baseline (week 0) use of acid-suppressive therapy. Participants receiving proton pump inhibitors or potassium-competitive acid blockers were classified as the PPI/P-CAB (+) group, whereas those receiving neither were classified as the PPI/P-CAB (−) group. To determine whether longitudinal changes in the gut microbiota differed with acid-suppressive therapy status, LEfSe analysis was performed separately in each subgroup, comparing gut microbiota profiles at baseline (week 0) and week 8 to identify bacterial taxa enriched after the intervention according to the criteria described above. In addition, to examine whether acid-suppressive therapy was associated with differences in gut microbiota composition after the intervention, LEfSe analysis was performed to compare week 8 gut microbiota profiles between the PPI/P-CAB (+) and PPI/P-CAB (−) groups. The laboratory personnel at the analysis facilities were blinded to the study time point and PPI/P-CAB status. These subgroup analyses were exploratory, and the findings were interpreted descriptively.

### 2.6. Safety Evaluation

Safety was evaluated throughout the study period by monitoring adverse events, vital signs, laboratory parameters, and clinically relevant findings. Adverse events of interest included rash, diarrhea, abdominal pain, flatulence, nausea, and other symptoms judged clinically relevant by the investigators.

### 2.7. Statistical Analysis

Continuous variables were summarized as median and interquartile range, and 95% CI, and categorical variables were given as number and percentage. Because most continuous variables were not normally distributed, changes from baseline (week 0) to week 8 were evaluated using the Wilcoxon signed-rank test. The primary endpoint was tested at a two-sided significance level of 5%. Because this was a single-arm study with multiple secondary endpoints, *p*-values for secondary endpoints were interpreted as exploratory and no formal adjustment for multiplicity was performed. Paired analyses were conducted using participants with evaluable data at baseline (week 0) and week 8 for each endpoint; therefore, the number of participants included in each analysis varied according to data availability. This exploratory interpretation also applied to subgroup analyses and gut microbiota analyses. Except for the predefined handling of missing daily BSFS values in the defecation score, missing endpoint data were not imputed. The main reasons for missing data were incomplete questionnaire responses, missing diary entries on defecation, missing laboratory measurements, and unavailable stool samples. No causal treatment effect was estimated in this single-arm design. No placebo-controlled comparison, formal adjustment for time-varying confounders, or post-intervention follow-up after week 8 was available; therefore, durability of the observed changes could not be evaluated.

## 3. Results

### 3.1. Study Population

**Figure 2:**
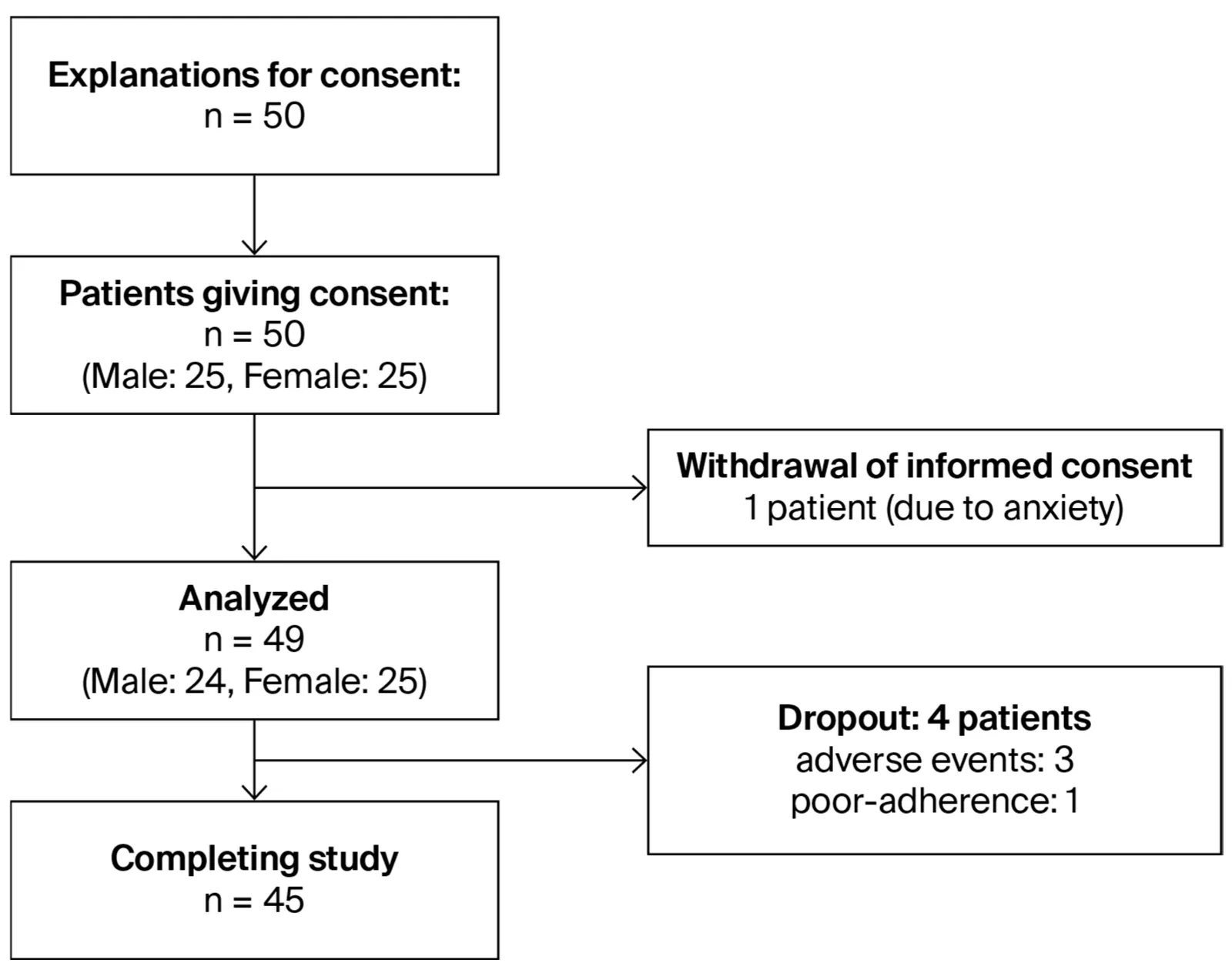
Study flow diagram. One patient was excluded due to withdrawal of informed consent; four dropped out due to abdominal pain (1), heartburn/burning sensation in the stomach/stomach pain (1), death (1), and poor adherence (1).

For the 50 participants who provided informed consent, 49 were included in the full analysis set after one participant discontinued the study at their own request. Overall, 45 participants completed the 8-week intervention. Four participants discontinued the study: three due to adverse events and one owing to poor compliance (Figure 2). Baseline characteristics are summarized in Table 1. A substantial proportion of participants were receiving acid-suppressive therapy, and nearly half had a history of laxative use. The patient characteristics at registry are shown in Table 1. Patients were registered from November 2021 to May 2022, and the study was terminated in July 2022. The paired primary endpoint analysis included 45 participants with evaluable defecation diary data at both baseline (week 0) and week 8. The medication adherence rate was 90%.

The number of participants included in each secondary endpoint analysis differed according to data availability, as shown in Supplementary Table S12.

### 3.2. Primary Endpoint

**Figure 3:**
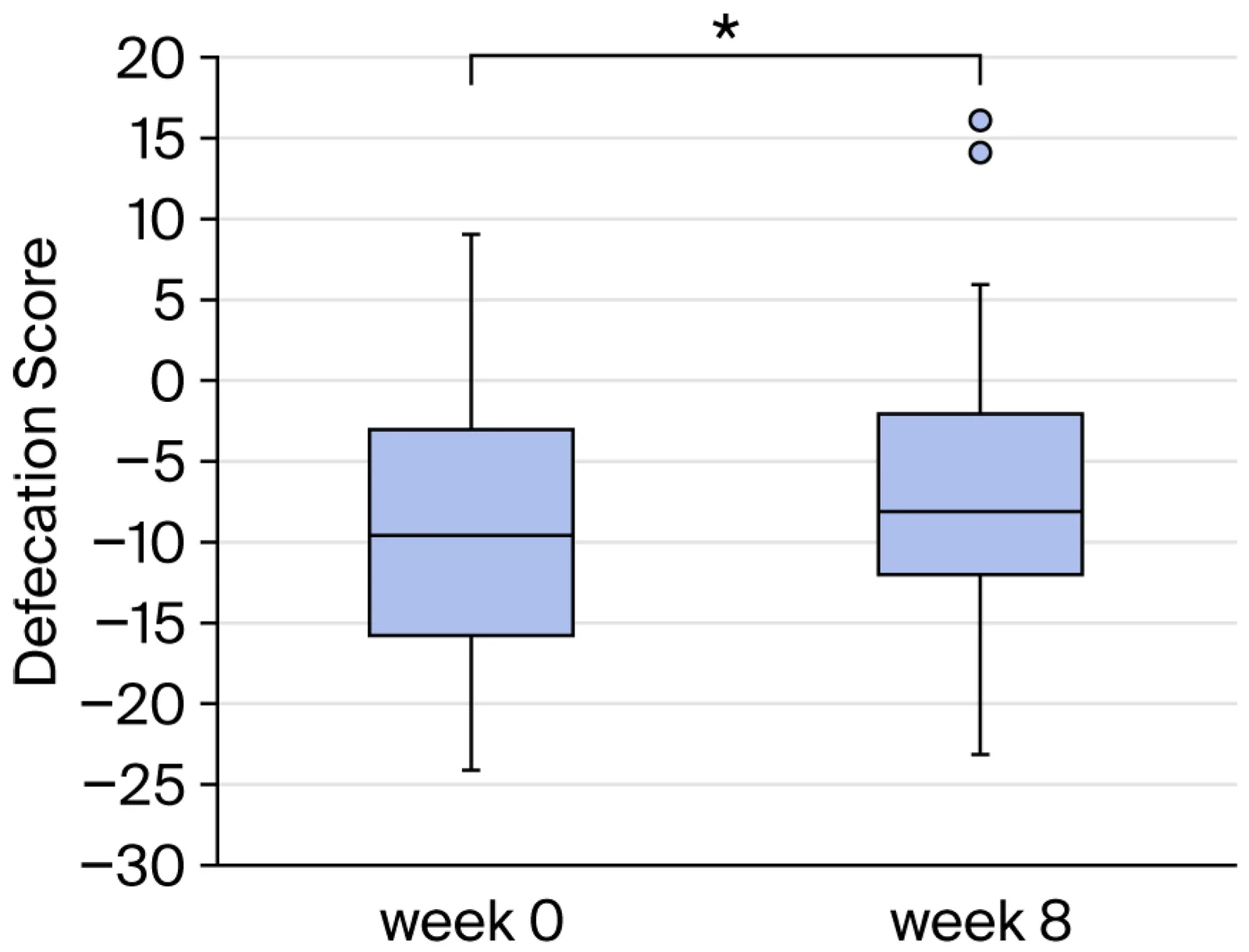
Changes in defecation score from baseline (week 0) to week 8. Statistical significance was assessed using the Wilcoxon signed-rank test. * *p* < 0.05.

For the primary endpoint, the defecation score based on the modified BSFS was assessed using the Wilcoxon signed-rank test and full analysis set (FAS). It showed a nominally significant change toward normalization of stool form from week 0 to week 8 in the overall study population (*p* = 0.024). Data are shown as box-and-whisker plots. The horizontal line within each box indicates the median, the box represents the interquartile range, and the whiskers indicate the range excluding outliers (Table 2, Figure 3).

For the sensitivity analysis, participants with missing data within the 7-day period (i.e., those with only 6 days of data) were excluded, and the results are shown in Supplementary Table S1.

In the exploratory subgroup analysis, participants were classified into the PPI/P-CAB (+) group or the PPI/P-CAB (−) group based on their use of acid-suppressive therapy (Table 3).

**Figure 4:**
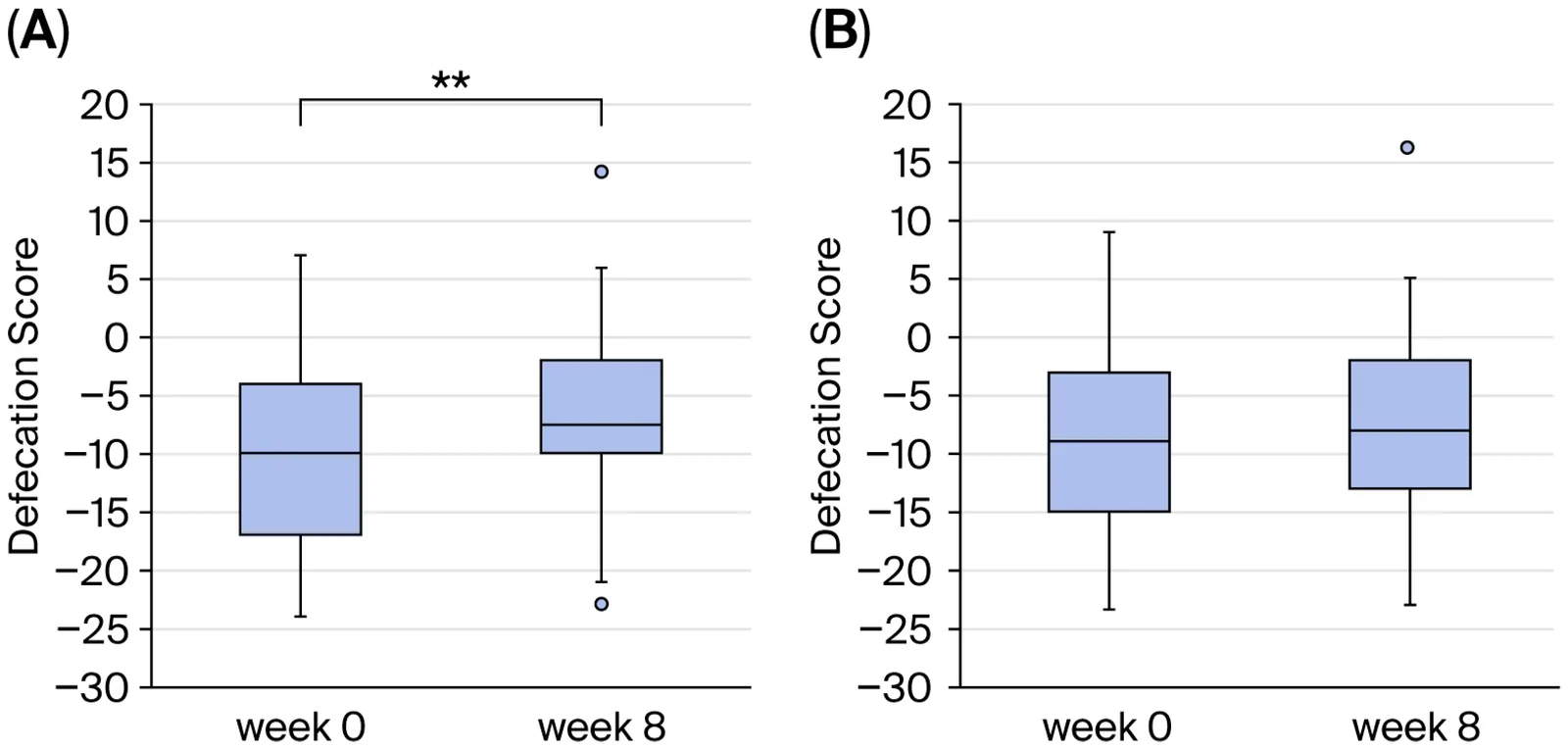
Changes in defecation score according to PPI/P-CAB use. (**A**) PPI/P-CAB (−) group (paired analysis, n = 22 at week 8). (**B**) PPI/P-CAB (+) group (paired analysis, n = 23 at week 8). Data are shown as box-and-whisker plots. Statistical significance was assessed using the Wilcoxon signed-rank test. ** *p* < 0.01.

The change in defecation score was larger in the PPI/P-CAB (−) group (*p* = 0.004), whereas no nominally significant change was observed in the PPI/P-CAB (+) group (*p* = 0.775) (Table 3, Figure 4). When comparing differences between both groups, the change in defecation score at week 8 was nominally greater in the PPI/P-CAB (−) group than in the PPI/P-CAB (+) group (*p* = 0.069).

As a sensitivity analysis was carried out, participants with missing data within the 7-day period (i.e., those with only 6 days of data) were excluded, and the results are shown in Supplementary Table S2.

Because this subgroup analysis was exploratory and not adjusted for multiplicity, these findings should be interpreted descriptively.

### 3.3. Secondary Endpoints

There was no significant change in stool frequency, evaluated as the 7-day average, from baseline (week 0) to week 8.

The straining severity score, also evaluated as the 7-day average, tended to improve at week 8 compared with the baseline (week 0), although this difference did not reach statistical significance (*p* = 0.057).

The change in the Bristol Stool Form Scale score from baseline (week 0) to week 8 was not nominally significant (*p* = 0.103). The change in the Bristol Stool Form Scale score appeared larger in the PPI/P-CAB (−) group (*p* = 0.030), whereas no nominally significant change was observed in the PPI/P-CAB (+) group (*p* = 0.933). When comparing differences between both groups, the change in Bristol Stool Form Scale score at week 8 was not nominally greater in the PPI/P-CAB (−) group than in the PPI/P-CAB (+) group (*p* = 0.128) (data are shown in Supplementary Table S3).

**Figure 5:**
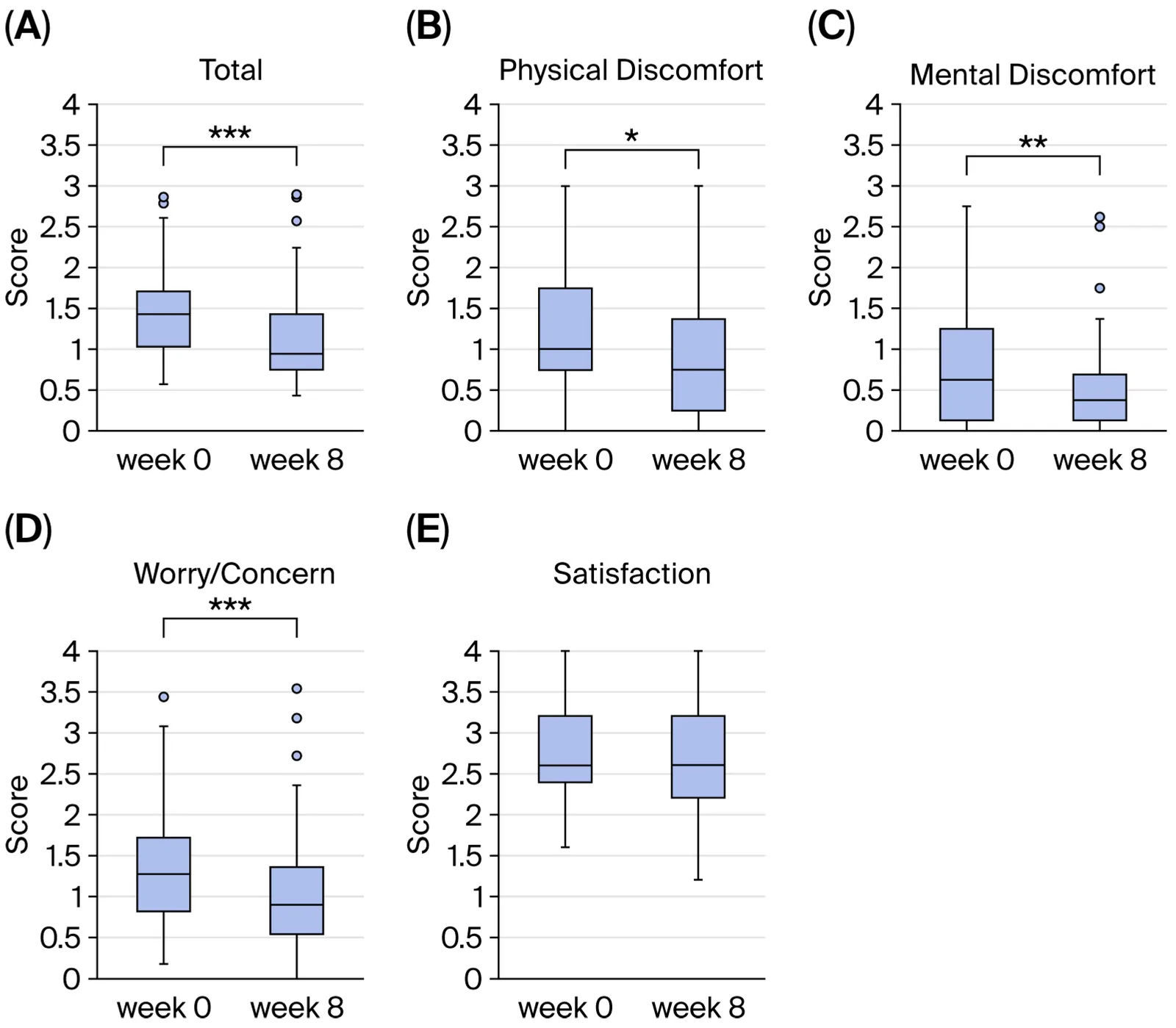
Changes in JPAC-QOL scores from baseline (week 0) to week 8. (**A**) Total score; (**B**) physical discomfort; (**C**) mental discomfort; (**D**) worry/concern; and (**E**) satisfaction. Data are shown as box-and-whisker plots. The horizontal line within each box indicates the median; the box represents the interquartile range; and the whiskers indicate the range excluding outliers. Statistical significance was assessed using the Wilcoxon signed-rank test. * *p* < 0.05, ** *p* < 0.01, *** *p* < 0.001. (Supplementary Table S4).

The JPAC-QOL total score showed a nominally significant decrease from baseline (week 0) to week 8, suggesting an association with improved constipation-specific quality of life (median change: −0.30; IQR: −0.50, 0.00; 95%CI: −0.42 and −0.08; *p* < 0.001) (Figure 5A). Among the JPAC-QOL subscales, nominally significant decreases were observed in physical discomfort (median change: −0.25; IQR: −0.50, 0.25; 95%CI: −0.40 and −0.02; *p* < 0.05) (Figure 5B), mental discomfort (median change: −0.13; IQR: −0.38, 0.00; 95%CI: −0.34 and −0.02; *p* < 0.01) (Figure 5C), and worry/concern (median change: −0.36; IQR: −0.82, 0.00; 95%CI: −0.51, −0.08; *p* < 0.01) (Figure 5D). These findings, based on secondary endpoints, should be interpreted cautiously because no multiplicity adjustment was performed. By contrast, no significant change was observed in the satisfaction subscale (Figure 5E). For more information, refer to Supplementary Table S4.

**Figure 6:**
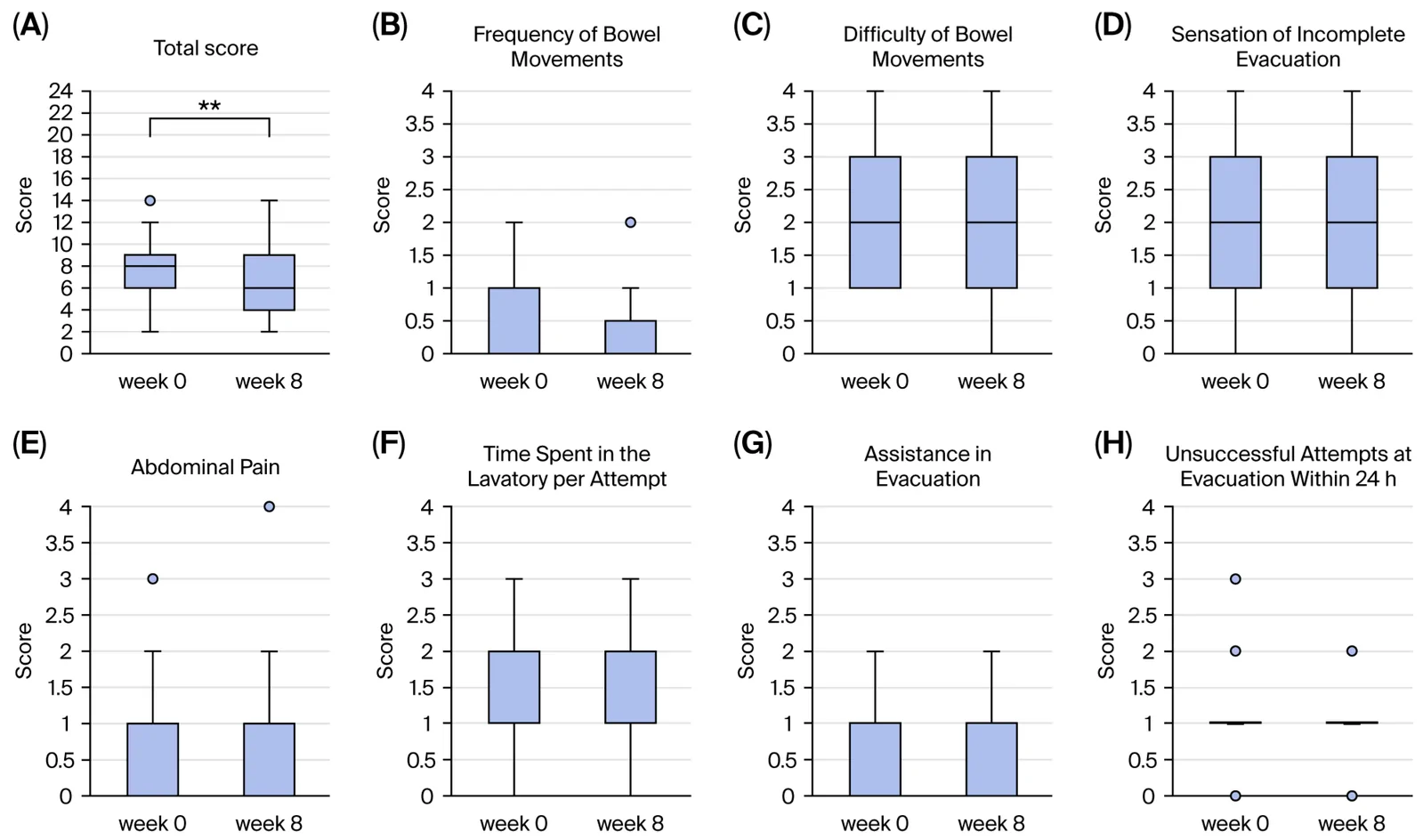
Changes in modified Constipation Scoring System scores from baseline (week 0) to week 8. (**A**) Total score; (**B**) frequency of bowel movements; (**C**) difficulty of bowel movements; (**D**) sensation of incomplete evacuation; (**E**) abdominal pain; (**F**) time spent in the lavatory per attempt; (**G**) assistance for evacuation; and (**H**) unsuccessful attempts at evacuation within 24 h. Data are shown as box-and-whisker plots. The horizontal line within each box indicates the median; the box represents the interquartile range; and the whiskers indicate the range excluding outliers. Statistical significance was assessed using the Wilcoxon signed-rank test. ** *p* < 0.01. (Supplementary Table S5).

Changes in the mCSS total score and subscale scores were evaluated from baseline (week 0) to week 8 (Figure 6 and Supplementary Table S5). The total mCSS score underwent a nominally significant decrease from baseline (week 0) to week 8, suggesting an association with lower constipation severity (median change: −1.5; IQR: −3.0, 0.0; 95%CI: −2.6 and −0.6; *p* < 0.01) (Figure 6A). Among the mCSS subscales, no significant changes were observed in bowel movement frequency, painful evacuation effort, sensation of incomplete evacuation, abdominal pain, time spent in the lavatory per attempt, assistance for evacuation, or unsuccessful attempts at evacuation within 24 h (Figure 6B–H). These results were exploratory and not adjusted for multiplicity.

**Figure 7:**
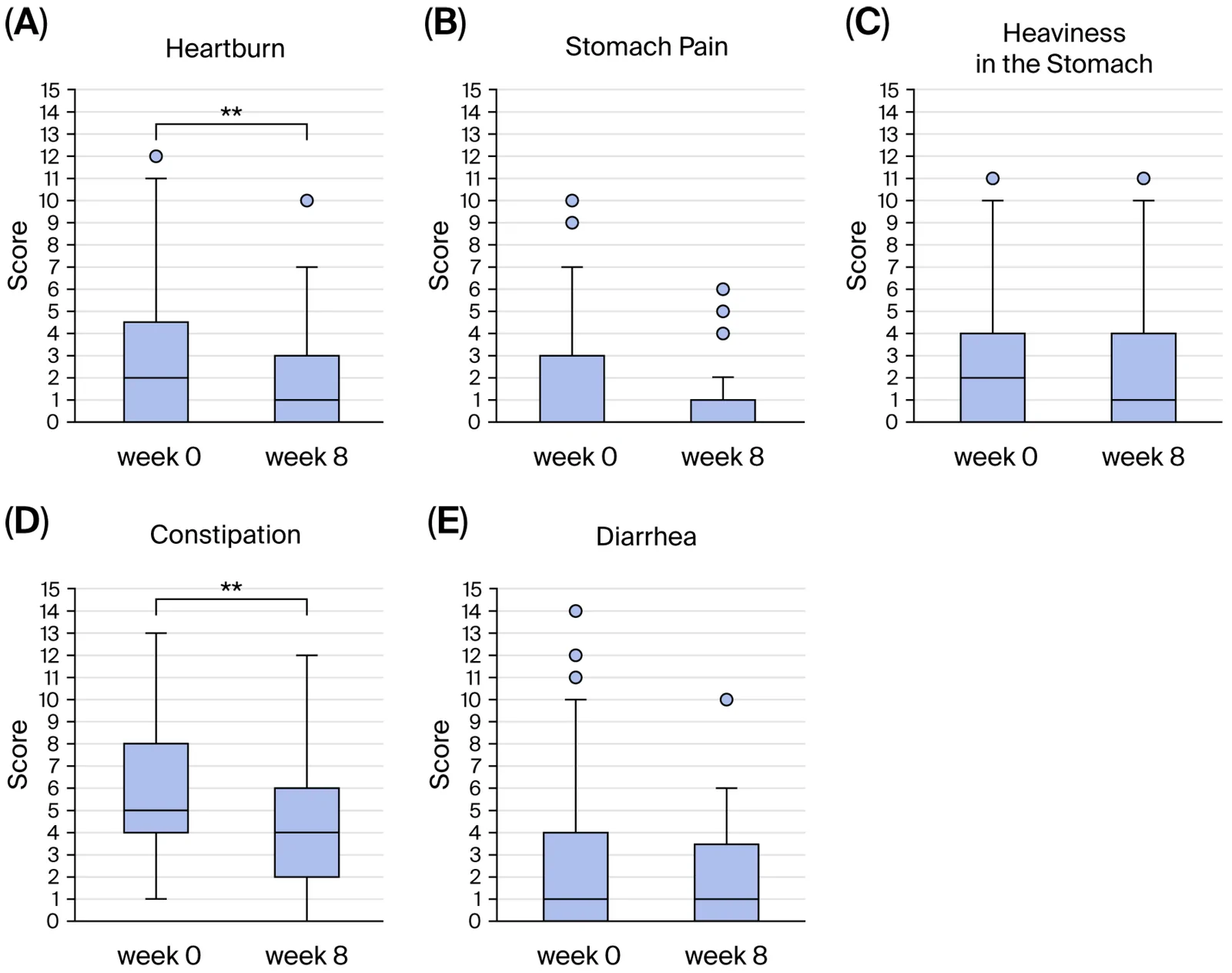
Changes in Izumo Scale scores from baseline (week 0) to week 8. (**A**) Heartburn, (**B**) stomach pain, (**C**) heaviness in the stomach, (**D**) constipation, and (**E**) diarrhea. Data are shown as box-and-whisker plots. The horizontal line within each box indicates the median; the box represents the interquartile range; and the whiskers indicate the range, excluding outliers. Statistical significance was assessed using the Wilcoxon signed-rank test. ** *p* < 0.01. (Supplementary Table S6).

For the Izumo Scale, the heartburn domain score showed a nominally significant decrease from baseline (week 0) to week 8, suggesting an association with lower reflux-related symptom burden (median change: 0.0; IQR: −2.0, 0.0; 95%CI: −0.54 and −0.08; *p* < 0.01) (Figure 7A). The constipation domain score also underwent a nominally significant decrease from baseline (week 0) to week 8 (median change: −1.0; IQR: −2.0, 0.0; 95%CI: −0.70 and −0.14; *p* < 0.01) (Figure 7D). In contrast, no significant changes were observed in the stomach pain, heaviness in the stomach, or diarrhea domains (Figure 7B,C,E). For more information, refer to Supplementary Table S6. These findings, based on secondary endpoints, should be interpreted cautiously because no multiplicity adjustment was performed.

**Figure 8:**
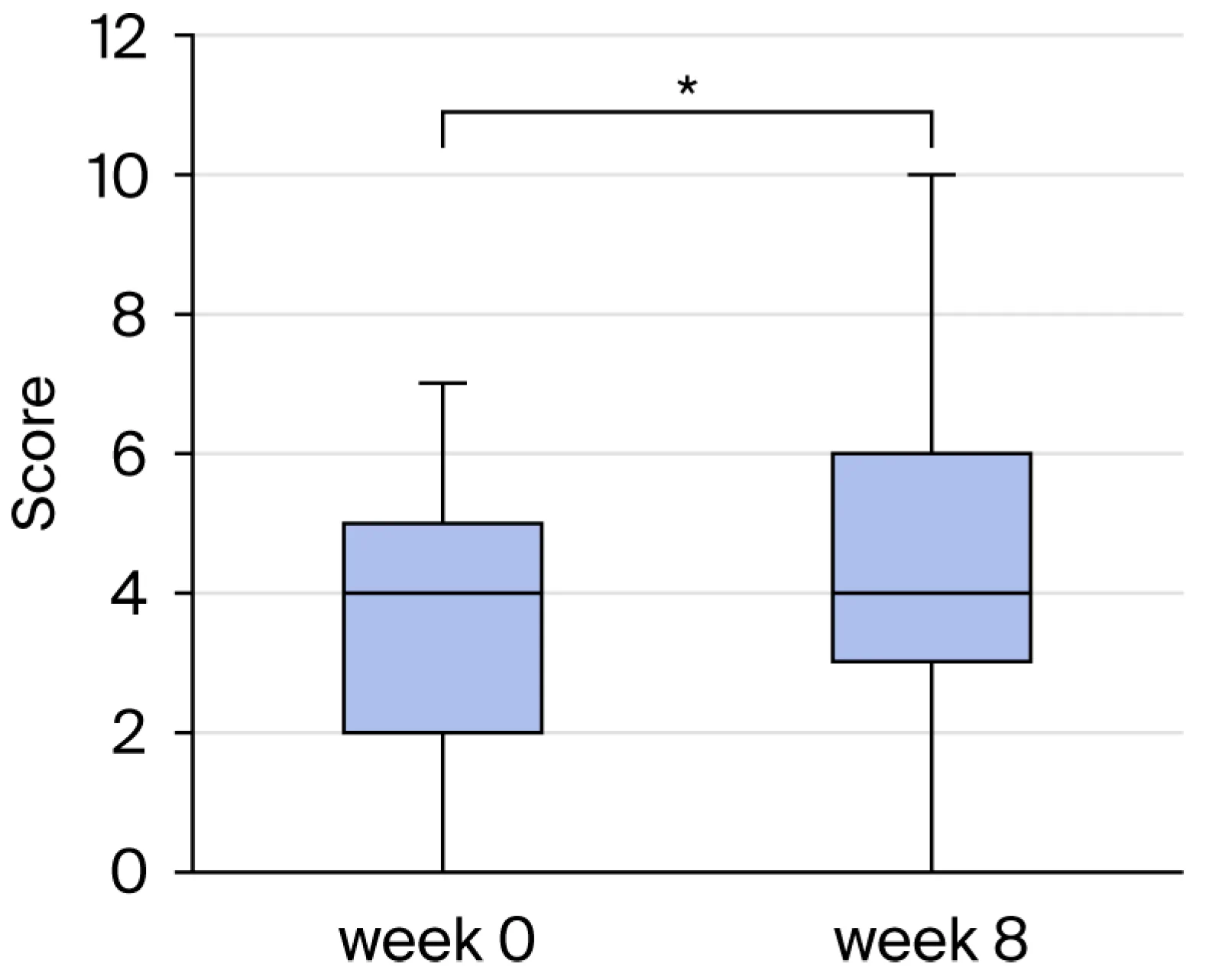
Changes in Dietary Variety Score from baseline (week 0) to week 8. Data are shown as box-and-whisker plots. The horizontal line within each box indicates the median; the box represents the interquartile range; and the whiskers indicate the range excluding outliers. Statistical significance was assessed using the Wilcoxon signed-rank test. * *p* < 0.05. (Supplementary Table S7).

The Dietary Variety Score (DVS) was compared between baseline (week 0) and week 8. A nominal change in the Dietary Variety Score (DVS) was observed from baseline (week 0) to week 8 (median change: 0.0; IQR: 0.0,1.0; 95%CI; 0.2 and 1.2; *p* < 0.05) (Figure 8, Supplementary Table S7).

**Figure 9:**
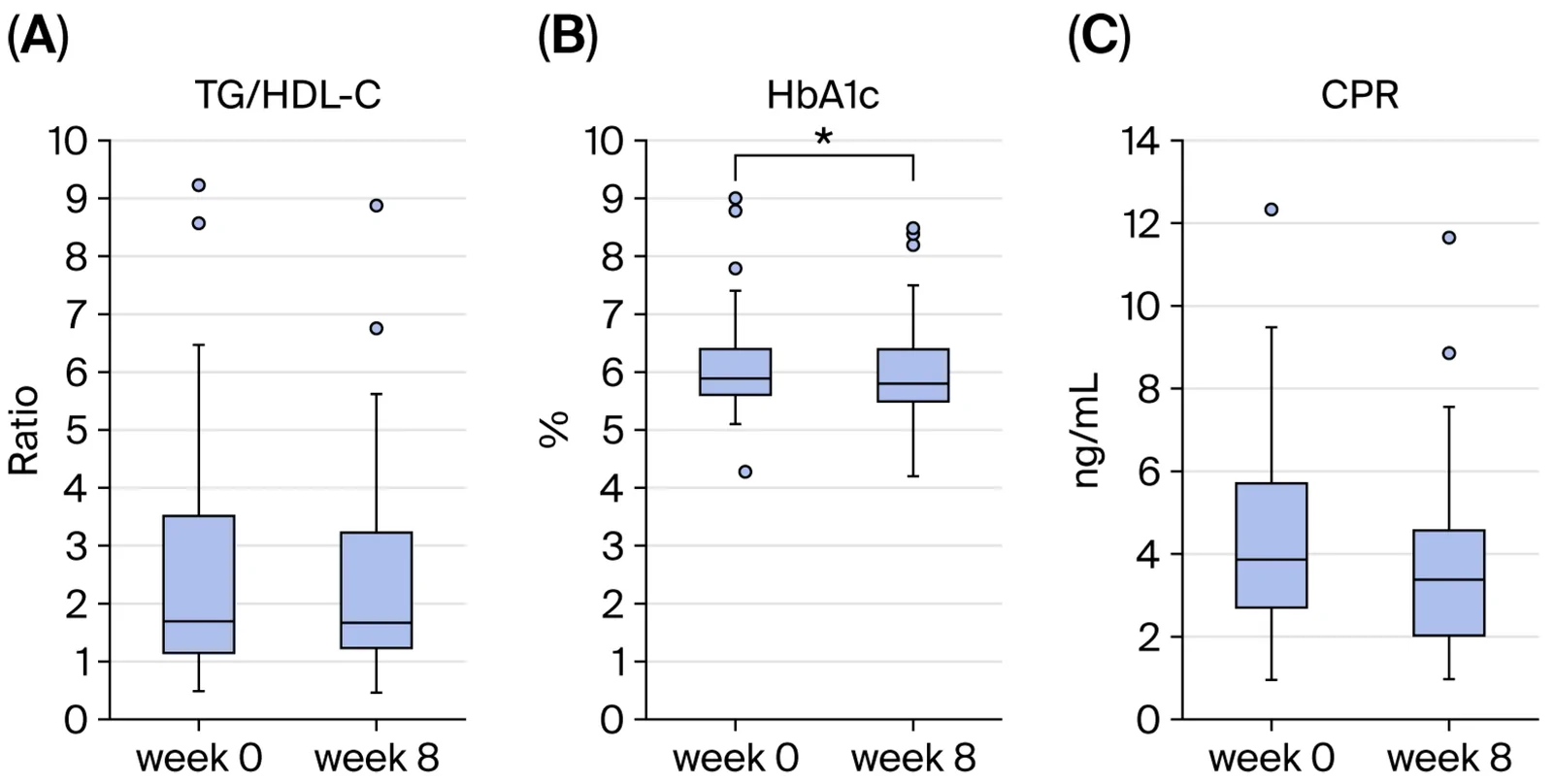
Changes in nutritional and glucose metabolism-related laboratory parameters from baseline (week 0) to week 8. (**A**) Triglyceride to high-density lipoprotein cholesterol ratio (TG/HDL-C ratio); (**B**) hemoglobin A1c (HbA1c); and (**C**) C-peptide immunoreactivity (CPR). A nominally significant decrease in HbA1c was observed from baseline (week 0) to week 8, and no significant changes were observed in the TG/HDL-C ratio or CPR. Data are shown as box-and-whisker plots. Statistical significance was assessed using the Wilcoxon signed-rank test. * *p* < 0.05. (Supplementary Table S7).

Changes in laboratory parameters related to nutritional status and glucose metabolism were evaluated from baseline (week 0) to week 8. Regarding nutritional assessment indices and glucose metabolism markers, no significant change was observed in the TG/HDL-C ratio from baseline (week 0) to week 8 (*p* = 0.243) (Figure 9A). In contrast, a nominally significant decrease in HbA1c was observed from baseline (week 0) to week 8 (median change: −0.10%; IQR: −0.15, −0.05; 95%CI: −0.15 and −0.01; *p* < 0.05) (Figure 9B). No significant change was observed in C-peptide immunoreactivity (CPR) (*p* = 0.137) (Figure 9C) (Supplementary Table S7).

Among vital signs, a nominally significant decrease in diastolic blood pressure was observed from baseline (week 0) to week 8 (median change: 0.0 mmHg; IQR: −6.0, 0.0; *p* = 0.032). No significant changes were observed in other laboratory parameters or vital signs. Similarly, no significant changes were observed in the GNRI, CONUT score, triglycerides, total cholesterol, or body weight index.

### 3.4. Gut Microbiota Findings

**Figure 10:**
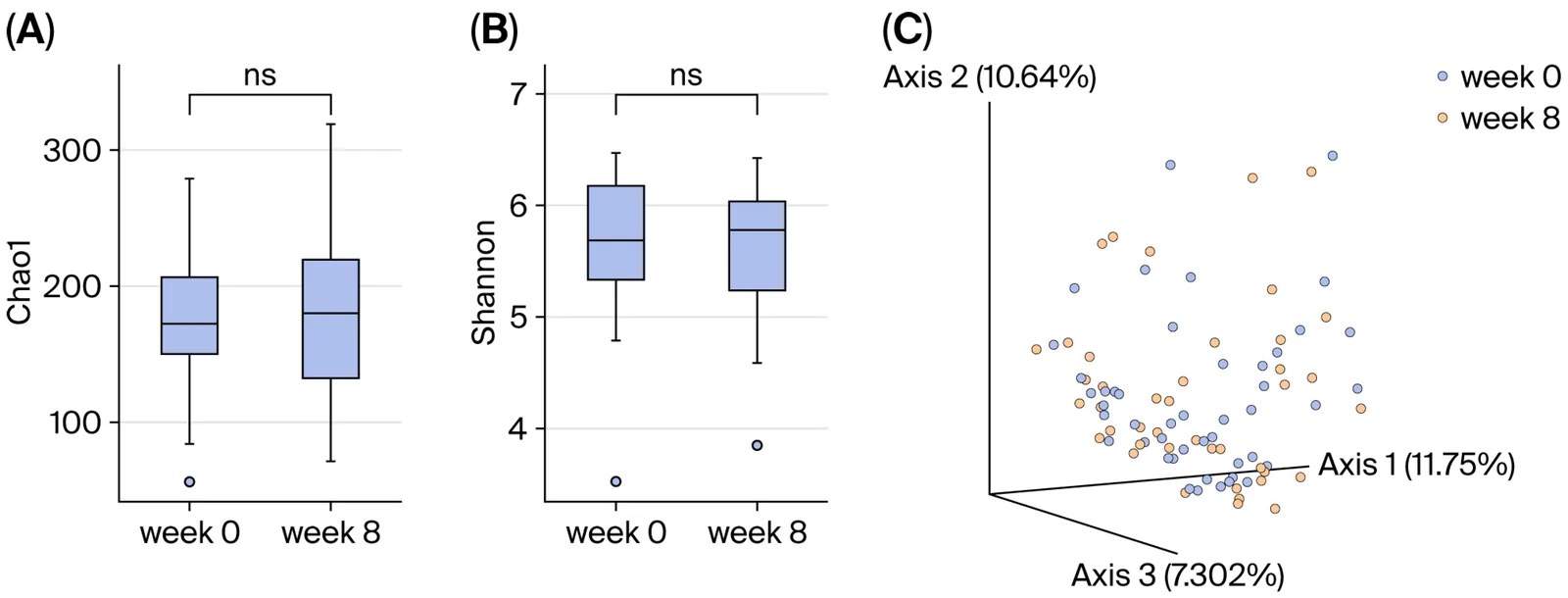
Changes in gut microbiota diversity from baseline (week 0) to week 8. (**A**) Chao1 richness index; (**B**) Shannon diversity index; and (**C**) principal coordinate analysis (PCoA) based on unweighted distance. No significant changes were observed in Chao1 richness or the Shannon diversity index from baseline (week 0) to week 8. PCoA was performed as an exploratory visualization of beta diversity. Statistical significance for alpha diversity indices was assessed using the Wilcoxon signed-rank test. ns, not significant (Supplementary Table S9).

Gut microbiota composition was evaluated using 16S rRNA amplicon sequencing of stool samples collected at baseline (week 0) and week 8. To understand changes in gut microbiota, stool samples were collected at two points: baseline (week 0) and week 8. Gut microbiota composition was analyzed using 16S rRNA amplicon sequencing. No significant changes were observed in alpha diversity indices, including Chao1 richness and the Shannon diversity index, from baseline (week 0) to week 8 (Figure 10A,B). Principal coordinate analysis (PCoA) based on unweighted distance was performed as an exploratory visualization of beta diversity; however, no apparent separation of gut microbiota profiles was observed between baseline (week 0) and week 8 (Figure 10C). Similarly, paired PERMANOVA revealed no significant changes in the overall microbiota structure (F = 0.234; R^2^ = 0.003; *p* = 0.958), and multivariate analysis of variance showed no significant differences in within-group variance across time points (F = 0.475, *p* = 0.534) (Supplementary Table S8).

The LEfSe analysis of the overall study population showed that no bacterial genera were significantly enriched at week 8 compared with baseline (week 0) using a predefined threshold of an LDA score >2.0 on a log10 scale and *p* < 0.05.

Because the impact of acid-suppressive therapy on gut microbiota composition and oral-to-gut microbial translocation is increasingly recognized [16,17,18], additional exploratory LEfSe analyses were performed according to PPI/P-CAB use status. Participants were classified into the PPI/P-CAB (+) group and the PPI/P-CAB (−) group.

**Figure 11:**
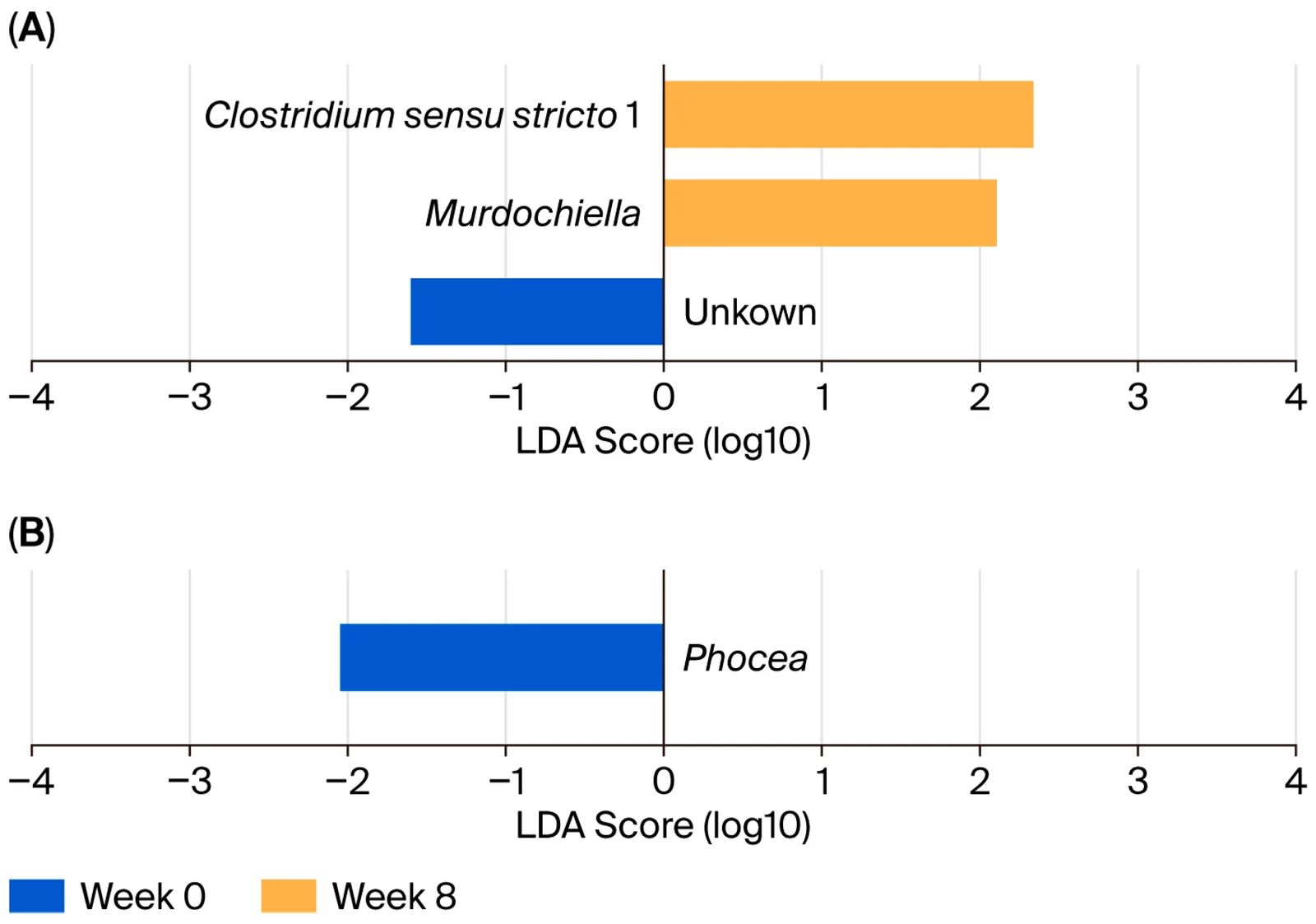
Exploratory LEfSe analysis of longitudinal changes in gut microbiota according to PPI/P-CAB use. (**A**) PPI/P-CAB (−) group (week 0, n = 23; week 8, n = 22). (**B**) PPI/P-CAB (+) group (week 0, n = 22; week 8, n = 21). LEfSe analysis was performed separately in the PPI/P-CAB (−) and PPI/P-CAB (+) groups to compare gut microbiota profiles between baseline (week 0) and week 8. In the PPI/P-CAB (−) group, *Clostridium sensu stricto* 1 and *Murdochiella* were nominally enriched at week 8 compared with the baseline (week 0). In the PPI/P-CAB (+) group, *Phocea* was nominally enriched at the baseline (week 0) compared with week 8, whereas no taxa were nominally enriched at week 8. Taxa were considered nominally enriched for exploratory purposes (with an LDA score > 2.0 on a log10 scale and *p* < 0.05) (Supplementary Table S10).

In the PPI/P-CAB (−) group, *Clostridium sensu stricto* 1, a genus that includes butyrate-producing bacteria, was nominally enriched at week 8 compared with the baseline (week 0) (LDA score: 2.338, *p* = 0.0222). *Murdochiella*, a genus whose physiological role in the human intestine remains unclear, was also nominally enriched at week 8 compared with the baseline (week 0) (LDA score: 2.103, *p* = 0.0381) (Figure 11A). In contrast, in the PPI/P-CAB (+) group, *Phocea* was nominally enriched at baseline (week 0) compared with week 8 (LDA score: 2.057, *p* = 0.0341), whereas no bacterial taxa were nominally enriched at week 8 (Figure 11B). These microbiota findings were exploratory and not adjusted for multiplicity (Supplementary Table S10).

**Figure 12:**
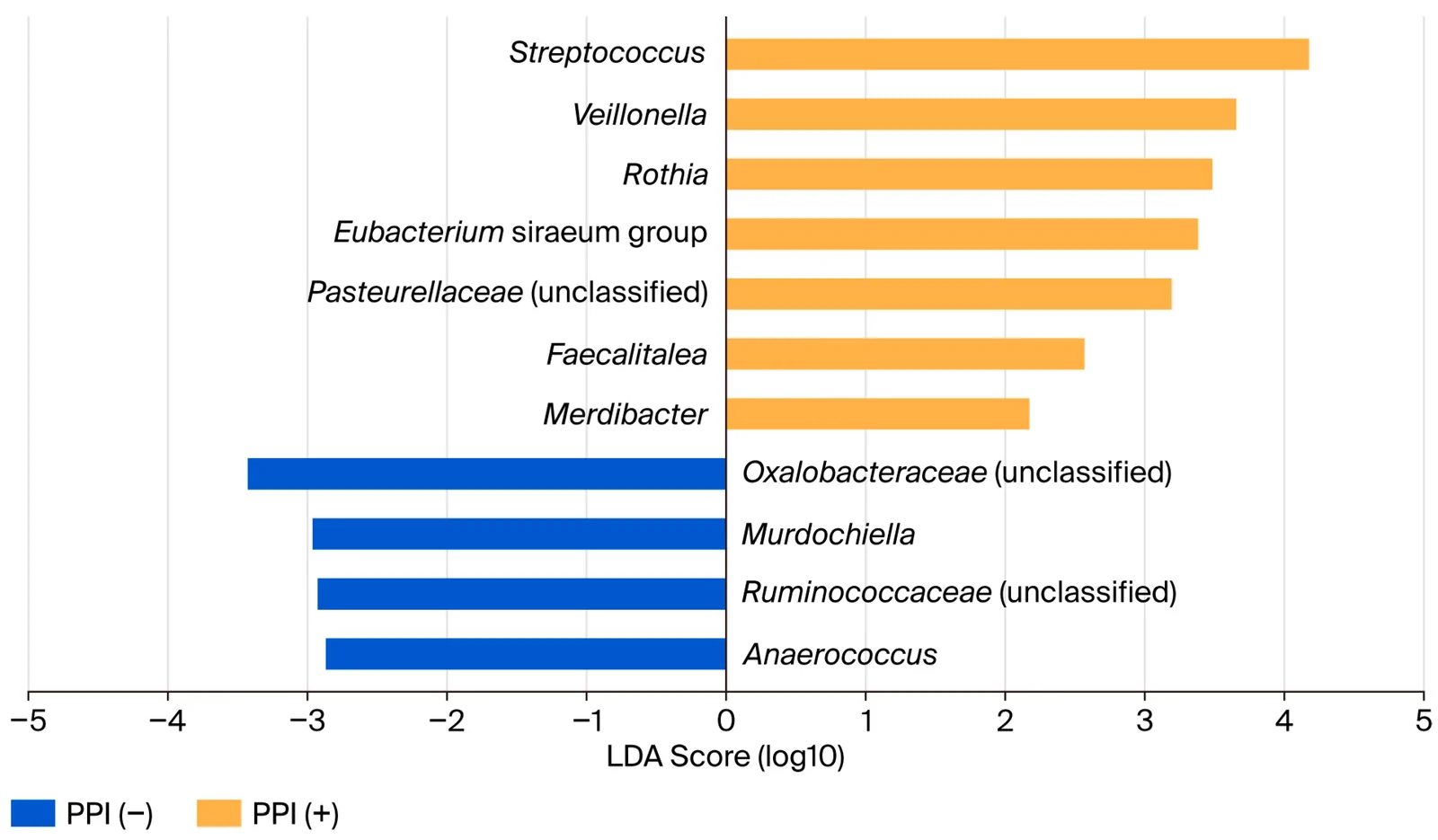
Exploratory LEfSe analysis comparing gut microbiota profiles according to PPI/P-CAB use at week 8.

Furthermore, LEfSe analysis was performed to compare gut microbiota profiles between the PPI/P-CAB (+) and PPI/P-CAB (−) groups at week 8 (Figure 12, Supplementary Table S11). Consistent with previous reports [19], the PPI/P-CAB (+) group showed significant enrichment of oral cavity-associated bacterial taxa, including *Streptococcus* (LDA score: 4.181, *p* = 0.0002), *Veillonella* (LDA score: 3.659, *p* < 0.0001), *Rothia* (LDA score: 3.485, *p* = 0.0319), and unclassified *Pasteurellaceae* (LDA score: 3.200, *p* = 0.0013). Taxa were considered nominally enriched for exploratory purposes (with an LDA score > 2.0 on a log10 scale and *p* < 0.05). These findings suggest that acid-suppressive therapy may be associated with distinct gut microbiota profiles characterized by enrichment of oral cavity-associated bacteria.

### 3.5. Safety

Table 4 summarizes the adverse events observed during the study period. Three adverse events occurred in three patients: abdominal pain in one patient, heartburn/a burning sensation in the stomach/stomach pain in one patient, and death due to an unexpected accident in one patient. All three patients withdrew from the study.

One serious adverse event occurred, namely death due to an unexpected accident; however, a causal relationship with the study drug was not confirmed. Among the mild adverse events, a causal relationship with the study drug could not be ruled out in two patients: abdominal pain occurred in one patient and heartburn/a burning sensation in the stomach/stomach pain in one patient.

## 4. Discussion

To the best of our knowledge, this is the first prospective interventional study in Japan to evaluate an orally disintegrating tablet containing three viable probiotic strains in older adults with functional constipation. In this single-center, open-label, single-arm study, 8 weeks of treatment was associated with changes in stool form and several patient-reported outcomes reflecting constipation burden. The intervention was generally well tolerated, with no clinically important safety concerns. Notably, the observed changes appeared to be associated with stool consistency and constipation-related symptoms rather than increased bowel frequency. This distinction is significant in older adults, for whom ease of defecation, relief of discomfort, and tolerability may be as meaningful as bowel frequency. Patient-reported outcomes supported this interpretation. The nominal changes in the JPAC-QOL total score and in the domains of physical discomfort, psychosocial discomfort, and worries/concerns suggest that the intervention may have been associated with a lower physical and perceived burden of constipation. These findings are broadly consistent with those of a previous single-arm prospective study by Fuyuki et al., in which 8 weeks of *Bifidobacterium bifidum* G9-1 administration improved the overall JPAC-QOL score and bowel movement frequency in patients with chronic constipation [24]. Similarly, changes in the total mCSS score suggest an overall reduction in constipation severity, although several individual subcomponents did not. However, these patient-reported outcomes were secondary endpoints and should be interpreted as exploratory because there was no adjustment for multiplicity. Microbiota profiling did not show a major global shift; however, the taxonomic findings provide hypotheses that may help explain the underlying mechanisms. Alpha diversity and exploratory beta diversity analyses showed no clear overall changes, which is plausible given the relatively short probiotic intervention. By contrast, LEfSe analysis suggested changes in *Clostridium sensu stricto* 1 after treatment among participants not receiving acid-suppressive therapy. Although species-level identification was not performed, this genus-level taxon includes *Clostridium butyricum* and related species; therefore, its enrichment may be related to administration of the *C. butyricum*-containing probiotic tablet [25]. Because this taxonomic group includes organisms associated with butyrate production and colonic homeostasis, the finding may be consistent with a constipation-improving mechanism. However, it remains exploratory and should not be interpreted as evidence of butyrate-mediated efficacy. *Murdochiella* has been detected as part of the mucosa-associated microbiota in the healthy human colon, particularly in the distal mucosa; however, its physiological role in the intestine remains unclear [26]. In the acid-suppressive therapy group, *Phocea* was nominally enriched at the baseline (week 0) and decreased by week 8, whereas no bacterial genus was nominally enriched at week 8. *Phocea* has been described as a human gut-associated genus, although its physiological relevance remains uncertain [27]. However, in ZDF fa/fa rats, the bacterial taxa that contributed most significantly to metabolic changes included *Phocea* [28].

The nominal enrichment of oral cavity-associated taxa in the PPI/P-CAB group is consistent with recent prospective evidence that proton pump inhibitors may alter the gut microbiota by facilitating oral-to-gut microbial translocation [29]. Several biologically plausible pathways may underlie the observed improvement in constipation symptoms. Yusuf et al. reported significantly lower fecal butyric acid levels in constipated older adults than in non-constipated controls [30]. The three-strain orally disintegrating tablet used in this study contains *Clostridium butyricum* TO-A, *Enterococcus faecium* T-110, and *Bacillus subtilis* TO-A, which is a combination that may have complementary effects on the intestinal environment and gut motility. Among these strains, *C. butyricum* TO-A has been shown to produce butyrate by utilizing lactate and acetate [31]. In addition, a probiotic preparation containing *B. subtilis* TO-A, *E. faecium* T-110, and *C. butyricum* TO-A increased fecal concentrations of butyric and acetic acids in a rat model of loperamide-induced constipation [6]. SCFAs, including acetate, propionate, and butyrate, may influence colonic motility and mucosal energy metabolism [32]. They may also activate GPR41 and GPR43 and promote the secretion of gut hormones such as peptide YY and glucagon-like peptide-1, both of which regulate intestinal motility [33,34]. The multispecies probiotic formulation may also be relevant. A meta-analysis of randomized controlled trials in adults with functional constipation showed that multispecies probiotics significantly improved bowel movement frequency and stool consistency; however, single-species probiotics did not show similar effects [4]. However, probiotic efficacy is strain- and disease-specific [35]. Therefore, the present findings are specific to this three-strain formulation and may not be generalized to other probiotic products. The findings related to acid-suppressive therapy may help explain heterogeneity in treatment response. Gastric acid suppression has been reported to alter the intestinal microbial environment and facilitate the translocation of oral-associated bacteria into the distal gut. In this study, oral cavity-associated taxa were nominally abundant in the PPI/P-CAB (+) group, whereas changes in defecation score appeared greater among participants not receiving acid-suppressive therapy. Acid-suppressive therapy, particularly with PPIs, has also been associated with gut dysbiosis, oral-to-gut bacterial translocation, and altered mucosal immune responses. These changes may partly explain differences in probiotic response according to acid-suppressive therapy status [36]. Because most available evidence concerns PPIs, the relevance of these findings to P-CABs should be interpreted cautiously. The observed nominal changes in heartburn should be interpreted conservatively and should not be considered direct evidence of an anti-reflux effect. A more plausible explanation is that changes in bowel status and overall gastrointestinal symptom burden reduced upper abdominal discomfort in participants; this is consistent with the known overlap among constipation, reflux symptoms, and dyspeptic complaints [37]. A similar improvement in upper abdominal symptoms has been reported in a probiotic intervention study in older adults with constipation [38]. The modest change in DVS may reflect a practical downstream consequence. As bowel-related discomfort, bloating, or concern about worsening constipation changes, patients may become more willing or able to maintain a broader diet. However, DVS is influenced by multiple behavioral factors and may also reflect routine variation, regression to the mean, or non-specific behavioral change. Similarly, the slight reduction in HbA1c is potentially interesting but exploratory. Although SCFAs can influence enteroendocrine pathways, including PYY and GLP-1 signaling, this study was not designed or powered to establish a metabolic effect [39]. Accordingly, all symptomatic and microbiota findings are interpreted strictly as uncontrolled pre–post associations rather than evidence of a causal therapeutic effect.

This study has several important limitations that substantially restrict the interpretation of efficacy. First, the single-center, open-label, single-arm design and relatively small sample size preclude exclusion of placebo effects, regression to the mean, expectation bias, reporting bias, and natural fluctuation of constipation symptoms. Thus, the present data cannot establish a causal relationship between the intervention and improvement in constipation. Second, external factors that may affect stool form and bowel habits were not adequately controlled. Diet, the quantity and quality of food intake, dietary fiber intake, fluid intake, physical activity level, and seasonal dietary changes were not systematically recorded or standardized, and any of these factors could have changed during the 8-week period and contributed to the observed results. Third, medications that may influence gastrointestinal motility were not comprehensively captured or adjusted for. Although selected medication histories, such as acid-suppressive therapy, prokinetic agents, aspirin, and laxatives, were summarized, anticholinergic agents, opioids, antidepressants, antihistamines, and other motility-affecting drugs were not systematically classified; this is particularly relevant in older adults with multimorbidity and polypharmacy. Fourth, comorbidities and organic causes of constipation were not excluded contemporaneously. Participants with known organic abnormalities detected by colonoscopy within the previous five years were excluded, but colonoscopy was not uniformly repeated immediately before enrollment. Therefore, undiagnosed polyps, diverticula, or active hemorrhoidal disease may have acted as confounders for constipation; for instance, in hemorrhoidal disease, patients may defecate less frequently for fear of worsening symptoms. Fifth, follow-up ended at week 8, and no post-intervention observation period was included. Consequently, the durability of the observed changes and whether they reflected sustained microbial changes, transient behavioral changes, or placebo-related responses could not be determined. Sixth, the primary endpoint was a modified stool form-based score rather than a conventional complete spontaneous bowel movement endpoint, and its clinical interpretation requires caution. Seventh, the number of evaluable participants differed across endpoints because of incomplete questionnaire responses, missing defecation diary data, missing laboratory measurements, or unavailable stool samples; complete-case paired analyses may therefore have introduced selection bias. Eighth, multiple secondary endpoints, subgroup analyses, and microbiota analyses were performed without formal multiplicity adjustment; therefore, nominally significant findings should be regarded as exploratory and hypothesis-generating. 

In addition, as the present study is exploratory, we did not set a power calculation when preparing the study protocol. Moreover, the rationale for selecting a target sample size of 50 participants was not verified by setting power to detect significance; instead, we referred to Nagamine, T et al. [13]. Therefore, for both of these reasons, the present study could have weak statistical power.

Finally, subgroup findings according to PPI/P-CAB exposure and LEfSe-based taxonomic enrichment were exploratory and susceptible to false-positive interpretation.

In each LEfSe comparison, 531 taxonomic clades were tested within relatively small subgroups without correction for multiple comparisons, which further increases this susceptibility. Moreover, conventional LEfSe does not adequately model paired longitudinal observations, and within-participant pairing across time points was not incorporated into analyses. We did not apply a differential abundance method developed for repeated-measures microbiome data, such as a mixed-effects model that treats participants as a random effect. The enriched taxa reported here should therefore be interpreted considering unadjusted descriptive signals that require confirmation in adequately powered and appropriately modeled studies. 16S rRNA profiling provides limited functional resolution; short-chain fatty acid concentrations, metabolomic profiles, and direct functional microbial pathways were not measured. Despite these limitations, this study provides pilot safety and feasibility data and generates hypotheses for future placebo-controlled randomized trials. It incorporates predefined confounder monitoring, systematic medication reviews, contemporaneous assessments of organic disease when clinically indicated, appropriate missing-data handling, multiplicity control, metabolomic integration, functional microbiome assessment, and post-intervention follow-up.

## 5. Conclusions

In this single-center, open-label, single-arm study, an orally disintegrating three-strain probiotic tablet appeared generally safe and well tolerated over 8 weeks in older adults with functional constipation. Although nominal pre–post changes in stool form and constipation-related symptom burden were observed, the absence of a control group, incomplete control of diet, fluid intake, physical activity, medications, and comorbidities, and the lack of post-intervention follow-up preclude causal conclusions and do not allow exclusion of placebo effects or other time-varying confounders. These findings should therefore be interpreted as exploratory pilot data that require confirmation in adequately powered randomized placebo-controlled trials with prespecified confounder monitoring and longer follow-up.

## Figures and Tables

**Table 1:** Patient characteristics at registry.

	Total	Without PPI	With PPI
Variable	Value		
Number of patients	49	23	26
Age, years	77.0 (71.0–83.0)	74 (69.0–78.0)	80.5 (75.0–84.0)
Sex			
Male	24 (49.0)	11 (47.8%)	13 (50.0%)
Female	25 (51.0)	12 (52.2%)	13 (50.0%)
Alcohol intake history			
No	33 (67.3)	14 (60.9%)	19 (73.1%)
Yes	16 (32.7)	9 (39.1%)	7 (26.9%)
Duration of alcohol intake, years	0.0 (0.0–20.0)	0.0 (0.0–20.0)	0.0 (0.0–20.0)
Smoking history			
No	32 (65.3)	13 (56.5%)	19 (73.1%)
Yes	17 (34.7)	10 (43.5%)	7 (26.9%)
Brinkman index	0.0 (0.0–600.0)	0.0 (0.0–750.0)	0.0 (0.0–400.0)
*H. pylori* infection status			
Negative	21 (42.9)	9 (39.1%)	12 (46.2%)
Positive	4 (8.2)	1 (4.3%)	3 (11.5%)
Negative after eradication	20 (40.8)	9 (39.1%)	11 (42.3%)
Unknown	4 (8.2)	4 (17.4%)	0 (0.0%)
History of PPI or P-CAB therapy			
No	23 (46.9)	23 (100.0%)	0 (0.0%)
PPI/ P-CAB	26 (53.1)	0 (0.0%)	26 (100.0%)
Aspirin intake history			
No	42 (85.7)	21 (91.3%)	21 (80.8%)
Yes	7 (14.3)	2 (8.7%)	5 (19.2%)
Prokinetic agent intake history			
No	40 (81.6)	20 (87.0%)	20 (76.9%)
Yes	9 (18.4)	3 (13.0%)	6 (23.1%)
Laxative intake history			
No	27 (55.1)	11 (47.8%)	16 (61.5%)
Yes	22 (44.9)	13 (52.2%)	10 (38.4)
Defecation-related variables			
Defecation score	−9.5 (−24.0, −9.0)	−10.0 (−17.0, −4.0)	−9.0 (−15.0, −3.0)
Bristol Stool Form Scale score	3.0 (2.0–4.0)	3.0 (2.0–4.0)	3.0 (2.0–4.0)
Duration of constipation, years n (%)			
0	9 (18.4)	3 (13.0)	6 (23.1)
1–5	18 (36.7)	9 (39.1)	9 (34.6)
6–10	7 (14.3)	4 (17.4)	3 (11.5)
11–20	5 (10.2)	2 (8.7)	3 (11.5)
≥21	8 (16.3)	5 (21.7)	3 (11.5)
Unknown	2 (4.1)	0 (0.0)	2 (7.7)
Body mass index, kg/m^2^	22.85 (20.7–24.9)	23.31 (21.22–25.39)	21.95 (20.31–24.36)
Laboratory data (reference values)			
White blood cell count, ×10^9^/L (3.6–8.9)	6.35 (5.15–7.00)	6030 (6.10–7.60)	6.40 (5.30–6.80)
Red blood cell count, ×10^12^/L (3.80–5.04)	4.13 (3.88–4.46)	4.17 (3.96–4.60)	4.08 (3.87–4.36)
Platelet count, ×10^9^/L (153.0–346.0)	205.0 (172.5–233.5)	202.0 (155.0–237.0)	208.0 (190.0–230.0)
Lymphocyte count, ×10^9^/L (153.0–346.0)	1.92 (1.57–2.44)	2.15 (1.73–2.53)	1.80 (1.36–2.02)
AST, U/L (5–37)	23.0 (18.0–26.0)	20.0 (18.0–30.0)	24.0 (19.0–26.0)
ALT, U/L (6–43)	16.0 (12.0–22.0)	17.0 (12.0–27.0)	15.0 (12.0–20.0)
γ-GTP, U/L (0–75)	22.0 (14.0–33.5)	22.0 (14.0–40.0)	20.0 (14.0–32.0)
Total protein, g/dL (6.5–8.5)	7.00 (6.7–7.2)	7.00 (6.60–7.20)	7.00 (6.80–7.20)
Albumin, g/dL (4–5.2)	4.20 (4.00–4.35)	4.20 (4.00–4.50)	4.10 (4.00–4.30)
Total cholesterol, mg/dL (150–219)	188.5 (167.0–210.5)	202.0 (146.0–216.0)	181.0 (167.0–206.0)
HDL cholesterol, mg/dL (40–70)	60.5 (52.5–72.0)	62.0 (49.0–75.0)	60.0 (68.0–68.0)
LDL cholesterol, mg/dL (70–139)	93.0 (77.7–113.5)	100.0 (72.0–118.0)	92.0 (77.0–106.0)
Triglycerides, mg/dL (30–149)	106.5 (85.0–188.5)	140.0 (90.0–232.0)	91.0 (79.0–120.0)
Fasting blood glucose, mg/dL (65–109)	112.5 (96.0–141.0)	118.0 (100.0–156.0)	105.0 (96.0–127.0)
HbA1c, % (4.6–6.2)	5.90 (5.60–6.40)	5.90 (5.60–6.80)	5.90 (5.70–6.20)
CPR, ng/mL (0.61–2.09)	3.87 (2.69–5.70)	3.86 (3.04–6.17)	3.87 (2.35–4.84)
CRP, mg/dL (0–0.3)	0.09 (0.03–0.16)	0.04 (0.03–0.10)	0.12 (0.05–0.24)

Values are presented as median (interquartile range) or n (%). PPI, proton pump inhibitor; P-CAB, potassium-competitive acid blocker; AST, aspartate aminotransferase; ALT, alanine aminotransferase; γ-GTP, gamma-glutamyl transpeptidase; HDL, high-density lipoprotein; LDL, low-density lipoprotein; CPR, C-peptide immunoreactivity; CRP, C-reactive protein.

**Table 2:** Changes in defecation score from baseline (week 0) to week 8.

Variable	Baseline (Week 0)	Week 8	Change from Baseline to Week 8	*p* Value
No. of patients	48	45	45	
Defecation score, median (IQR) 95% CI	−9.5 (−15.7, −3.0) −11.9, −6.8	−8.0 (−12.0, −2.0) −9.6, −4.2	2.0 (−1.0, 7.0) 0.0, 3.8	0.024

**Table 3:** Changes in defecation score according to PPI/P-CAB use.

Defecation Score		Week 0	n	Week 8	n	Amount of Change	n	*p* Value
PPI/P-CAB (−)	Median (IQR) 95% CI	−10 (−17, −4) −14.0, −6,2	23	−7.5 (−10, −2) −10.8, −2.9	22	4 (0, 7) 0.8, 5.6	22	0.004
PPI/P-CAB (+)	Median (IQR) 95% CI	−9 (−15, −3.0) −12.1, −5.0	25	−8 (−13, −2) −11.0, −2.9	23	0 (−4, −6) −2.4, 3.7	23	0.775

**Table 4:** Adverse events during the study period.

Adverse Event	No. of Patients, n (%)	No. of Events
Total adverse events	3 (6.1)	3
Abdominal pain	1 (2.0)	1
Heartburn/burning sensation in the stomach/stomach pain	1 (2.0)	1
Death due to an unexpected accident	1 (2.0)	1

## Data Availability

The data presented in this study are available on request from the corresponding author.

## Supplementary Materials

The following supporting information can be downloaded at https://www.mdpi.com/article/10.3390/diseases14090317/s1. Supplementary Table S1. Defecation score (total); Supplementary Table S2. Defecation score (PPI/P-CAB +) (PPI/P-CAB −); Supplementary Table S3. Bowel movement score on the Bristol Stool Form Scale; Supplementary Table S4. JPAQ-QOL score; Supplementary Table S5. mCSS score; Supplementary Table S6. Izumo Scale score; Supplementary Table S7. DVS, TG/HDL-C, HbA1c, CPR; Supplementary Table S8. paired PERMANOVA; Supplementary Table S9. Changes in gut microbiota diversity from baseline (week 0) to week 8 (Chao1 and Shannon index); Supplementary Table S10. Exploratory LEfSe analysis of longitudinal changes in gut microbiota according to PPI/P-CAB use (LDA score); Supplementary Table S11. Exploratory LEfSe analysis comparing gut microbiota profiles according to PPI/P-CAB use at week 8 (LDA score). Supplementary Table S12. Trends in the Number of Cases.
