# Supplementary material for: Safety and Exploratory Pre–Post Changes Associated with an Orally Disintegrating Three-Strain Probiotic Tablet in Older Adults with Functional Constipation: A Single-Center, Open-Label, Single-Arm Study
Source: Diseases. 2026 Aug 31;14(9):317. doi: 10.3390/diseases14090317 (PMC13605162; doi:10.3390/diseases14090317)
Supplement: Supplementary file 1 [file diseases-14-00317-s001.zip › diseases-4445049-supplementary.pdf]

Supplementary Table S1. Defecation score (Total) (Sensitivity analysis)

		week 0	n	week 8	n	amount of change	n	*p*-value
Defecation score	Median (IQR)	-9.0(-15.0, -3.0)	46	-8.0(-12.0, -2.0)	45	1.5（-1.5, 6.5）	44	0.036
	95%CI	-11.4, -6.3		-9.6 -4.2		-0.2, -3.7		

Missing data were excluded. The weekly total of defecation score. *Signed-rank Wilcoxon test

Supplementary Table S2. Defecation score (PPI/P-CAB +) (PPI/P-CAB -) (Sensitivity analysis)

Defecation score		week 0	n	week 8	n	amount of change	n	*p*-value	Comparison between groups (*p*-value, 95%CI)
PPI/P-CAB (-)	Median (IQR)	-10.0(-17, -4)	23	-7.5(-10, -2)	22	4.0(0.0, 7.0)	22	0.004	0.039 (-0.9, 6.7)
	95%CI	-14.0, -6.2		-10.8, -2.9		0.8, 5.6			
PPI/P-CAB (+)	Median (IQR)	-9.0(-15, -2.0)	23	-8.0(-13, -2)	22	0.0(-4.0, -3.0)	22	1.000	
	95%CI	-11.1, -4,0		-11.0, -2.9		-2.8, 3.4			

Missing data were excluded. The weekly total of defecation score by PPI/P-CAB use. *Signed-rank Wilcoxon test

Supplementary Table S3. Bowel movement score on the Bristol Stool Form Scale

Bristol Stool Form Scale		week 0	n	week 8	n	amount of change	n	*p*-value	Comparison between groups (*p*-value, 95%CI)
PPI/P-CAB (-)	Median (IQR)<br>95%CI	3.3(2.1, 4.7)<br>2.8, 4.0	23	3.7(3.0, 4.0)<br>3.2, 4.2	22	0.5(0.0, 1.0)<br>0.0, 0.8	22	0.030	0.128<br>(-0.3, 0.9)
PPI/P-CAB (+)	Median (IQR)<br>95%CI	3.6(2.6, 4.3)<br>3.1, 4.0	25	3.9(3.0, 4.4)<br>3.1, 4.2	23	-0.2(-0.5, 0.8)<br>-0.4, 0.6	23	0.933	
Total population	Median (IQR)<br>95%CI	3.6(2.4, 4.3)<br>3.1, 3.8	48	3.7(3.0, 4.3)<br>3.3, 4.0	45	0.3(-0.4, 0.8)<br>-0.1, 0.6	45	0.103	–

The weekly average of stool form scores (ranging from 1 to 7). *Signed-rank Wilcoxon test

Supplementary Table S4. JPAC-QOL Score

JPAC-QOL		week 0	n	week 8	n	amount of change	n	*p*-value
Total	Median (IQR)<br>95%CI	1.43(1.04, 1.71)<br>1.27, 1.62	43	0.95(0.75, 1.43)<br>1.00, 1.41	40	-0.30(-0.50, 0.00)<br>-0.42, - 0.08	34	<0.001
Physical discomfort	Median (IQR)<br>95%CI	1.00(0.75, 1.75)<br>1.02, 1.42	48	0.75(0.25 ,1.38)<br>0.73, 1.27	44	-0.25(-0.50, 0.25)<br>-0.40, -0.02	43	0.029
Mental discomfort	Median (IQR)<br>95%CI	0.63(0.13, 1.25)<br>0.57, 0.96	47	0.38(0.13, 0.69)<br>0.36, 0.74	44	-0.13(-0.38, 0.00)<br>-0.34, -0.02	42	0.005
Worry/ concern	Median (IQR)<br>95%CI	1.27(0.82, 1.73)<br>1.17, 1.62	46	0.91(0.55, 1.36)<br>0.82, 1.34	42	-0.36(-0.82, 0.00)<br>-0.51, -0.08	39	0.002
Satisfactory	Median (IQR)<br>95%CI	2.60(2.40, 3.20)<br>2.51, 2.91	46	2.60(2.20, 3.20)<br>2.43, 2.87	42	-0.20(-0.60, 0.40)<br>-0.33, 0.14	39	0.289

Supplementary Table S5. mCSS score

mCSS		week 0	n	week 8	n	amount of change	n	*p*-value
Total	Median (IQR)<br>95%CI	8.0(6.0, 9.0)<br>7.0, 8.7	41	6.0(4.0, 9.0)<br>5.8, 7.9	42	-1.5(-3.0, 0.0)<br>-2.6, -0.6	34	0.002
Frequency of bowel movements	Median (IQR)<br>95%CI	0.0(0.0, 1.0)<br>0.2, 0.6	48	0.0(0.0, 0.5)<br>0.1, 0.4	44	0.0(0.0, 0.0)<br>-0.3, 0.1	43	0.307
Difficulty of bowel movements	Median (IQR)<br>95%CI	2.0(1.0, 3.0)<br>1.9, 2.4	48	2.0(1.0, 3.0)<br>1.6, 2.3	44	0.0(-1.0, 0.0)<br>-0.6, 0.2	43	0.274
Sensation of incomplete evacuation	Median (IQR)<br>95%CI	2.0(1.0, 3.0)<br>1.7, 2.3	48	2.0(1.0, 3.0)<br>1.4, 2.0	43	0.0(-1.0 ,0.0)<br>-0.7, 0.1	42	0.182

Abdominal pain	Median (IQR)<br>95%CI	1.0(0.0, 1.0)<br>0.4, 0.9	47	0.0(0.0, 1.0)<br>0.3, 0.8	44	0.0(-1.0, 0.0)<br>-0.4, 0.1	42	0.176
Time spent in the lavatory per attempt	Median (IQR)<br>95%CI	1.0(1.0, 2.0)<br>1.1, 1.5	46	1.0(1.0, 2.0)<br>1.1, 1.5	43	0.0(0.0, 0.0)<br>-0.3, 0.1	40	0.367
Assistance in evacuation	Median (IQR)<br>95%CI	0.0(0.0, 1.0)<br>0.2, 0.6	47	0.0(0.0, 1.0)<br>0.2, 0.5	44	0.0(0.0, 0.0)<br>-0.3, 0.1	42	0.364
Unsuccessful attempts at evacuation within 24 hours	Median (IQR)<br>95%CI	1.0(1.0, 1.0)<br>0.7, 1.1	45	1.0(1.0, 1.0)<br>0.8, 1.1	44	0.0(0.0, 0.0)<br>-0.2, 0.2	40	1.000

Supplementary Table S6. IZUMO Scale score

IZUMO scale		week 0	n	week 8	n	amount of change	n	*p*-value
Heartburn	Median (IQR)<br>95%CI	2.0(0.0, 4.5)<br>0.65, 1.34	48	1.0(0.0, 3.0)<br>0.36, 0.88	44	0.0(-2.0, 0.0)<br>-0.54, -0.08	43	0.008
Stomach pain	Median (IQR)<br>95%CI	0.0(0.0, 3.0)<br>0.32, 0.85	48	0.0(0.0, 1.0)<br>0.13, 0.45	44	0.0(-1.0, 0.0)<br>-0.54, -0.00	43	0.067
Heaviness in the stomach	Median (IQR)<br>95%CI	2.0(0.0, 4.0)<br>0.56, 1.12	48	1.0(0.0, 4.0)<br>0.47, 1.16	44	0.0(-2.0, 2.0)<br>-0.31, 0.20	43	0.724
Constipation	Median (IQR)<br>95%CI	5.0(4.0, 8.0)<br>1.64, 2.21	48	4.0(2.0, 6.0)<br>1.21, 1.88	44	-1.0(-2.0, 0.0)<br>-0.70, -0.14	43	0.004
Diarrhea	Median (IQR)<br>95%CI	1.0(0. 0, 4.0)<br>0.49, 1.22	48	1.0(0.0, 3.5)<br>0.39, 0.85	44	0.0(-1.0, 1.0)<br>-0.57, 0.06	43	0.206

Supplementary Table S7. DVS, TG/HDL-C, HbA1c, CPR

		week 0	n	week 8	n	amount of change	n	*p*-value
DVS (score)	Median (IQR)<br>95%CI	4.0(2.0, 5.0)<br>3.1, 4.2	47	4.0(3.0, 6.0)<br>3.9, 5.2	44	0.0(0.0, 1.0)<br>0.2, 1.2	42	0.014
TG/HDL-C（mg/dL）	Median (IQR)	1.69(1.14, 3.51)	48	1.65(1.21, 3.21)	45	-0.19(-0.76, 0.45)	44	0.243
HbA1c（%）	Median (IQR)<br>95%CI	5.9(5.6,6.4)<br>5.90, 6.10	48	5.8(5.5,6.4)<br>5.80, 6.32	45	-0.10(-0.15,0.05)<br>-0.15, -0.01	44	0.025
CPR	Median (IQR)	3.87(2.69, 5.70)	48	3.37(2.03, 4.58)	45	-0.08(-1.58, 0.81)	44	0.137

Supplementary Table S8 Changes in gut microbiota β diversity from baseline to week 8(PERMANOVA index)

(n=43)

PERMANOVA			Within-group variance	
F value	R2 value	P value	F value	P value
0.234	0.003	0.958	0.475	0.534

This analysis covered 43 samples for which corresponding data from Week 0 and Week 8 were available.

Supplementary Table S9. Changes in gut microbiota α diversity from baseline to week 8 (Chao1 and Shannon index)

		week 0	n	week 8	n	*p*-
chao1	Median (IQR)	176(150, 205)	43	180(132 ,220)	4	0.923
Shannon	Median (IQR)	5.68(5.35, 6.18)	43	5.78(5.24, 6.03)	4	0.575

n:sample number.

This analysis covered 43 samples for which corresponding data from Week 0 and Week 8 were available.

Supplementary Table S10. Exploratory LEfSe analysis of longitudinal changes in gut microbiota according to PPI/P-CAB use (LDA Score)

	Bacteria	Day 0	Day 8	*p*-value
PPI /P-CAB (-)	*Clostridium sensu* stricto 1	-	2.338	0.0222
	Murdochiella	-	2.103	0.0381
	Unknown Bacteria	-1.604	-	0.0381
PPI/P-CAB (+)	*Phocea*	-2.057	-	0.0341

LDA (Linear Discriminant Analysis)

Supplementary Table S11. Exploratory LEfSe analysis comparing gut microbiota profiles according to PPI/P-CAB use at week 8 (LDA Score)

	Bacteria	Week 8	*p*-value
PPI/P-CAB (+)	*Streptococcus*	4.181	0.0002
	Veillonella	3.659	<0.0001
	Rothia	3.485	0.0319
	*Eubacterium sira*eum group	3.379	0.0461
	Pasteurellaceae (unclassified)	3.196	0.0013
	Faecalitalea	2.571	0.0218
	Merdibacter	2.170	0.0370
PPI /P-CAB (-)	*Oxalobacteraceae* (unclassified)	-3.422	0.0092
	Murdochiella	-2.962	0.0381
	Ruminococcaceae (unclassified)	-2.926	0.0453
	Anaerococcus	-2.866	0.0189

LDA (Linear Discriminant Analysis)

Supplementary Table S12. Trends in the Number of Cases

(n)

		IC	FAS	week 0	week 8
Defecation score	Total	50	49	48	45
	PPI/P-CAB (-)	50	49	23	22
	PPI/P-CAB (+)	50	49	25	23
JPAC-QOL	Total	50	49	43	40
	Physical discomfort	50	49	48	44
	Mental discomfort	50	49	47	44
	Worry/ concern	50	49	46	42
	Satisfactory	50	49	46	42
mCSS	Total	50	49	41	42
	Frequency of bowel movements	50	49	48	44
	Difficulty of bowel movements	50	49	48	44
	Sensation of incomplete evacuation	50	49	48	43
	Abdominal pain	50	49	47	44
	Time spent in the lavatory per attempt	50	49	46	43
	Assistance in evacuation	50	49	47	44
	Unsuccessful attempts at evacuation within 24	50	49	45	44
IZUMO scale	Heartburn	50	49	48	44
	Stomach pain	50	49	48	44
	Heaviness in the stomach	50	49	48	44
	Constipation	50	49	48	44

	Diarrhea	50	49	48	44
DVS (score)		50	49	47	44
TG/HDL-C（mg/dL）		50	49	48	45
HbA1c（%）		50	49	48	45
CPR		50	49	48	45
chao1		50	49	43	43
Shannon		50	49	43	43
PCoA		50	49	43	43
LEfSe analysis		50	49	45	43
